# Semisynthesis and Pesticidal Activities of Novel Cholesterol Ester Derivatives Containing Cinnamic Acid-like Fragments

**DOI:** 10.3390/molecules27238437

**Published:** 2022-12-02

**Authors:** Rongfei Lu, Jianwei Xu, Zhen Wang, Shaoyong Zhang, Hailong Wang, Hui Xu, Min Lv

**Affiliations:** 1 College of Plant Protection, Northwest A & F University, Xianyang 712100, China; 2 Key Laboratory of Vector Biology and Pathogen Control of Zhejiang Province, College of Life Science, Huzhou University, Huzhou 313000, China; 3 Yan’an Institute of Agricultural Sciences, Yan’an 716000, China

**Keywords:** cholesterol, cinnamic acid, structural modification, agricultural activity

## Abstract

Due to the extensive use of agrochemicals resulting in the emergence of pesticide resistance and ecological environment problems, the research and development of new alternatives for crop protection is highly desirable. In order to discover potent natural product-based insecticide candidates, a series of new cholesterol ester derivatives containing cinnamic acid-like fragments at the C-7 position were synthesized. Some derivatives showed potent pesticidal activities. Against *Mythimna separata* Walker, compounds **2a**, **Id**, **Ig**, and **IIg** showed 2.1–2.4-fold growth-inhibitory activity of the precursor cholesterol. Against *Plutella xylostella* Linnaeus, compounds **Ig**, **IIf**, and **IIi** exhibited 1.9–2.1-fold insecticidal activity of cholesterol. These results will pave the way for the future synthesis of cholesterol-based derivatives as agrochemicals.

## 1. Introduction

**Figure 1:**
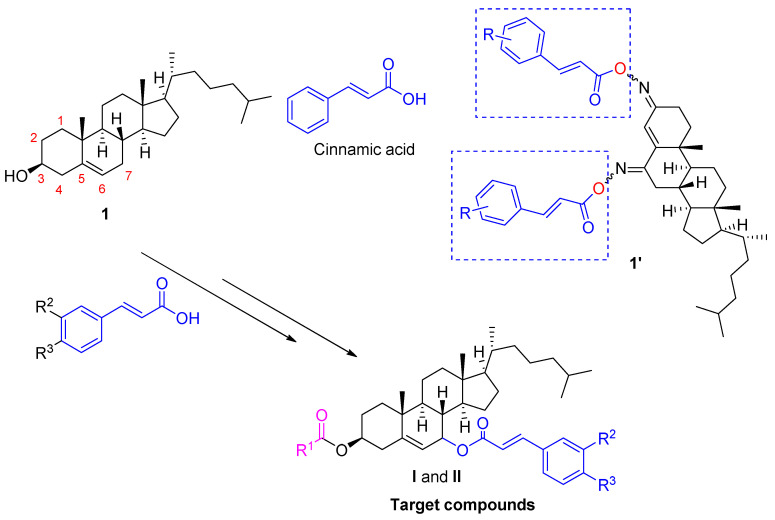
Design of cholesterol esters derivatives **I** and **II**.

Cholesterol (**1**), a biological endogenous substance, has shown a variety of antitumor, antioxidant, anti-inflammatory, and insecticide activities [1,2,3,4,5,6,7,8,9,10]. Cinnamic acid (Figure 1), as a main component of *Cinnamomum cassia* Presl, has displayed a range of pharmaceutical and agricultural activities [11,12,13]. In addition, several cholesterol oxime esters containing cinnamic acid-like fragments (**1′**) have shown promising insecticidal activities [14]. Nowadays, due to the extensive and irrational use of chemical insecticides resulting in pest resistance and ecological environment problems, the research and development of new pesticides for crop protection is highly desirable [15,16,17,18]. Because of the characteristics of natural secondary metabolites as an unparalleled source of bioactive products, currently, the study of natural product-based insecticides as an important alternative to classic agrochemicals has received much attention [19,20,21]. Therefore, in order to develop prospective cholesterol-based pesticide candidates, a series of new cholesterol derivatives **I** and **II** (Figure 1) were designed and synthesized by a combination of cholesterol and cinnamic acid-like fragments via the ester bond at the C-7 position of cholesterol. Meanwhile, two typically crop-threatening insect pests, *Mythimna separata* Walker (Lepidoptera: Noctuidae) and *Plutella xylostella* Linnaeus (Lepidoptera: Plutellidae), were used as the tested biotargets for assessment of the insecticidal activities of cholesterol derivatives **I** and **II** [22,23].

## 2. Materials and Methods

Multi-generation sensitive strains of *Mythimna separata* and *Plutella xylostella* (3rd instar larvae) were raised in our lab. Cholesterol was purchased from Aladdin Chemistry Co., Ltd. (Shanghai, China). The melting point (mp) was determined using the XT-415 digital melting point apparatus (Beijing Tech Instruments, Ltd., Beijing, China) and was uncorrected. ^1^H NMR spectra (Supplementary Materials) were obtained using Avance III 500 MHz equipment (Bruker, Germany). High-resolution mass spectra (HRMS) were obtained with a MicrOTOF Q II instrument (Bruker, Germany). Infrared spectroscopy (IR) was obtained with a TENSOR-27 instrument (Bruker, Germany).

### 2.1. Synthesis of Compounds ***2a*** and ***2b***

A mixture of cholesterol (**1**, 5 mmol), triethylamine (10 mmol), and substituted acyl chloride (7 mmol) in dry CH_2_Cl_2_ (20 mL) was stirred at room temperature for 14 or 17 h. Then, CH_2_Cl_2_ (30 mL) was added to the mixture, which was washed with brine. The organic phase was dried over anhydrous Na_2_SO_4_ and purified by silica gel column chromatography eluting with petroleum ether/dichloromethane (4/1, *v*/*v*) to obtain **2a** and **2b**.

*Data for* ***2a***: CAS: 604-32-0. Yield: 79%, white solid, mp 188–190 °C; IR cm^−1^ (KBr): 2948, 1717, 1656, 1531, 1454, 1370, 1271, 1110, 710; ^1^H NMR (500 MHz, CDCl_3_) *δ*: 8.05 (d, *J* = 7.5 Hz, 2H, Ar-H), 7.56 (t, *J* = 7.5 Hz, 1H, Ar-H), 7.44 (t, *J* = 7.5 Hz, 2H, Ar-H), 5.42 (d, *J* = 5.0 Hz, 1H, H-6), 4.83–4.89 (m, 1H, H-3), 2.47 (d, *J* = 8.5 Hz, 2H), 1.97–2.03 (m, 3H), 1.90–1.93 (m, 1H), 1.80–1.86 (m, 1H), 1.69–1.78 (m, 1H), 1.45–1.61 (m, 7H), 1.33–1.38 (m, 3H), 1.09–1.27 (m, 8H), 1.07 (s, 3H), 0.99–1.02 (m, 2H), 0.93 (d, *J* = 6.5 Hz, 3H), 0.87 (s, 3H), 0.86 (s, 3H), 0.69 (s, 3H). HRMS (ESI): calcd for C_34_H_50_O_2_Na ([M + Na]^+^), 513.3616; found, 513.3709.

*Data for* ***2b***: CAS: 30591-44-7. Yield: 89%, white solid, mp 180–182 °C; IR cm^−1^ (KBr): 2949, 2859, 1710, 1464, 1276, 1112, 750; ^1^H NMR (500 MHz, CDCl_3_) *δ*: 7.93 (d, *J* = 8.0 Hz, 2H, Ar-H), 7.23 (d, *J* = 8.0 Hz, 2H, Ar-H), 5.42 (d, *J* = 5.0 Hz, 1H, H-6), 4.81–4.87 (m, 1H, H-3), 2.46 (d, *J* = 8.0 Hz, 2H), 2.40 (s, 3H), 1.97–2.03 (m, 3H), 1.89–1.92 (m, 1H), 1.82–1.85 (m, 1H), 1.68–1.76 (m, 1H), 1.46–1.59 (m, 7H), 1.33–1.38 (m, 3H), 1.09–1.27 (m, 8H), 1.06 (s, 3H), 0.99–1.02 (m, 2H), 0.92 (d, *J* = 6.5 Hz, 3H), 0.87 (s, 3H), 0.86 (s, 3H), 0.68 (s, 3H). HRMS (ESI): calcd for C_35_H_52_O_2_Na ([M + Na]^+^), 527.3865; found, 527.3862.

### 2.2. Synthesis of Compounds ***3a*** and ***3b***

A mixture of compounds **2a** and **2b** (4 mmol), CrO_3_ (4 mmol), pyridine (8 mmol), and 70% *t*-BuOOH (40 mmol) in dry CH_2_Cl_2_ (30 mL) was stirred at room temperature for 5 or 12 h. The mixture was extracted by CH_2_Cl_2_ (30 mL × 2). The combined organic phase was washed with saturated aq. NaHSO_3_ (15 mL × 3) and brine (15 mL), dried over anhydrous Na_2_SO_4_ and purified by silica gel column chromatography eluting with petroleum ether/dichloromethane (1/3, *v*/*v*), yielded **3a** and **3b**.

*Data for* ***3a***: CAS: 6997-41-7. Yield: 93%, white solid, mp 158–160 °C; IR cm^−1^ (KBr): 2944, 2866, 1717, 1667, 1459, 1275, 1113, 712; ^1^H NMR (500 MHz, CDCl_3_) *δ*: 8.05 (d, *J* = 7.5 Hz, 2H, Ar-H), 7.58 (t, *J* = 7.5 Hz, 1H, Ar-H), 7.46 (t, *J* = 7.5 Hz, 2H, Ar-H), 5.75 (s, 1H, H-6), 4.94–5.01 (m, 1H, H-3), 2.60–2.72 (m, 2H), 2.38–2.43 (m, 1H), 2.23–2.28 (m, 1H), 2.11–2.14 (m, 1H), 2.01–2.06 (m, 2H), 1.79–1.94 (m, 2H), 1.48–1.61 (m, 5H), 1.34–1.39 (m, 5H), 1.26 (s, 3H), 1.07–1.15 (m, 6H), 1.01–1.04 (m, 1H), 0.93 (d, *J* = 6.5 Hz, 3H), 0.87 (s, 3H), 0.86 (s, 3H), 0.69 (s, 3H). HRMS (ESI): calculated for C_34_H_48_O_3_Na ([M + Na]^+^), 527.3506; found, 527.3501.

*Data for* ***3b***: Yield: 74%, white solid, mp 161–162 °C; IR cm^−1^ (KBr): 2949, 2865, 1715, 1667, 1461, 1278, 1112, 746; ^1^H NMR (500 MHz, CDCl_3_) *δ*: 7.93 (d, *J* = 7.5 Hz, 2H, Ar-H), 7.24 (d, *J* = 8.0 Hz, 2H, Ar-H), 5.74 (s, 1H, H-6), 4.92–4.99 (m, 1H, H-3), 2.58–2.71 (m, 2H), 2.41 (s, 3H), 2.23–2.27 (m, 1H), 2.09–2.14 (m, 1H), 2.00–2.06 (m, 2H), 1.89–1.94 (m, 1H), 1.78–1.83 (m, 1H), 1.48–1.59 (m, 4H), 1.27–1.39 (m, 7H), 1.25 (s, 3H), 1.06–1.18 (m, 6H), 1.00–1.04 (m, 1H), 0.93 (d, *J* = 6.5 Hz, 3H), 0.87 (d, *J* = 2.0 Hz, 3H), 0.86 (d, *J* = 2.0 Hz, 3H), 0.69 (s, 3H). HRMS (ESI): calculated for C_35_H_50_O_3_Na ([M + Na]^+^), 541.3658; found, 541.3659.

### 2.3. Synthesis of Compounds ***4a*** and ***4b***

Compound 3a or b (4 mmol) was dissolved in methanol/dichloromethane (1/1, *v*/*v*), and sodium borohydride (6 mmol) was added in batches under an ice bath. Then, it was raised naturally to room temperature. When the reaction was complete, methanol was removed and ethyl acetate (30 mL) was added to the mixture, which was washed with brine. The organic phase was dried over anhydrous Na_2_SO_4_, concentrated, and purified by silica gel column chromatography eluting with petroleum ether/dichloromethane (1/1, *v*/*v*) to obtain **4a** and **4b**.

*Data for* ***4a***: CAS: 17974-80-0. Yield: 65%, white solid, mp 192–193 °C; IR cm^−1^ (KBr): 3490, 2946, 2859, 1691, 1456, 1282, 1122, 708; ^1^H NMR (500 MHz, CDCl_3_) *δ*: 8.05 (d, *J* = 7.5 Hz, 2H, Ar-H), 7.56 (t, *J* = 7.5 Hz, 1H, Ar-H), 7.45 (t, *J* = 7.5 Hz, 2H, Ar-H), 5.36 (s, 1H, H-6), 4.85–4.91 (m, 1H, H-3), 3.89 (d, *J* = 8.0 Hz, 1H, H-7), 2.48–2.50 (m, 2H), 2.02–2.04 (m, 2H), 1.86–1.93 (m, 2H), 1.70–1.84 (m, 2H), 1.28–1.58 (m, 12H), 1.14–1.22 (m, 6H), 1.11 (s, 3H), 0.98–1.04 (m, 1H), 0.93 (d, *J* = 6.5 Hz, 3H), 0.88 (d, *J* = 2.5 Hz, 3H), 0.86 (d, *J* = 2.0 Hz, 3H), 0.70 (s, 3H). HRMS (ESI): calculated for C_34_H_50_O_3_Na ([M + Na]^+^), 529.3645; found, 529.3658.

*Data for* ***4b***: Yield: 68%, white solid, mp 189–191 °C; IR cm^−1^ (KBr): 3483, 2948, 2861, 1688, 1285, 1121, 1026, 751; ^1^H NMR (500 MHz, CDCl_3_) *δ*: 7.93 (d, *J* = 8.0 Hz, 2H, Ar-H), 7.23 (d, *J* = 8.0 Hz, 2H, Ar-H), 5.35 (s, 1H, H-6), 4.83–4.89 (m, 1H, H-3), 3.89 (d, *J* = 8.0 Hz, 1H, H-7), 2.46–2.49 (m, 2H), 2.40 (s, 3H), 1.99–2.04 (m, 2H), 1.86–1.92 (m, 2H), 1.72–1.82 (m, 2H), 1.30–1.56 (m, 12H), 1.12–1.21 (m, 6H), 1.11 (s, 3H), 1.00–1.04 (m, 1H), 0.93 (d, *J* = 6.5 Hz, 3H), 0.87 (d, *J* = 2.5 Hz, 3H), 0.86 (d, *J* = 2.5 Hz, 3H), 0.70 (s, 3H). HRMS (ESI): calculated for C_35_H_52_O_3_Na ([M + Na]^+^), 543.3814; found, 543.3814.

### 2.4. General Procedure for Synthesis of Target Compounds ***I**(**a**–**i**)–**II**(**a**–**i**)*

Compound **4a** or **4b** (0.4 mmol) reacted with cinnamic acids (**5a**–**i**, 0.6 mmol) in dry CH_2_Cl_2_ (5 mL) in the presence of EDCI (0.6 mmol) and DMAP (0.2 mmol) at room temperature for 10–34 h. Then, the mixture was diluted with CH_2_Cl_2_ (20 mL). It was washed with brine, dried over anhydrous Na_2_SO_4_, and purified by PTLC eluting with petroleum ether/dichloromethane (*v*/*v* = 4:1) or petroleum ether/ethyl acetate (*v*/*v* = 15:1) to afford **I**(**a**–**i**)–**II**(**a**–**i**) in 14−53% yields.

*Data for****Ia***: Yield: 18%, white solid, mp 105–106 °C; IR cm^−1^ (KBr): 2946, 2863, 1712, 1634, 1497, 1451, 1266, 1244, 1269, 1110, 710; ^1^H NMR (500 MHz, CDCl_3_) *δ*: 8.04 (d, *J* = 8.0 Hz, 2H, Ar-H), 7.59 (d, *J* = 16.0 Hz, 1H, H-3′), 7.52–7.54 (m, 1H, Ar-H), 7.41–7.44 (m, 2H, Ar-H), 7.00–7.04 (m, 2H, Ar-H), 6.81 (d, *J* = 8.0 Hz, 1H, Ar-H), 6.26 (d, *J* = 16.0 Hz, 1H, H-2′), 6.00 (s, 2H, –OCH_2_O–), 5.35 (s, 1H, H-6), 5.20 (d, *J* = 8.5 Hz, 1H, H-7), 4.85–4.91 (m, 1H, H-3), 2.49 (d, *J* = 8.5 Hz, 2H), 2.03–2.06 (m, 2H), 1.92–1.96 (m, 1H), 1.73–1.83 (m, 3H), 1.47–1.59 (m, 5H), 1.30–1.36 (m, 4H), 1.19–1.24 (m, 4H), 1.16 (s, 3H), 1.06–1.13 (m, 4H), 0.97–1.01 (m, 1H), 0.92 (d, *J* = 6.5 Hz, 3H), 0.86 (d, *J* = 2.0 Hz, 3H), 0.84 (d, *J* = 2.0 Hz, 3H), 0.72 (s, 3H). HRMS (ESI): calculated for C_44_H_56_O_6_Na ([M + Na]^+^), 703.3975; found, 703.3979.

*Data for****Ib:*** Yield: 21%, white solid, mp 106–107 °C; IR cm^−1^ (KBr): 2944, 2864, 1711, 1636, 1507, 1459, 1280, 1243, 1167, 708; ^1^H NMR (500 MHz, CDCl_3_) *δ*: 8.04 (d, *J* = 7.5 Hz, 2H, Ar-H), 7.52–7.57 (m, 2H), 7.44 (t, *J* = 7.5 Hz, 2H, Ar-H), 7.02–7.05 (m, 2H, Ar-H), 6.85 (d, *J* = 8.0 Hz, 1H, Ar-H), 6.28 (d, *J* = 15.5 Hz, 1H, H-2´), 5.36 (s, 1H, H-6), 5.20 (d, *J* = 8.5 Hz, 1H, H-7), 4.85–4.91 (m, 1H, H-3), 4.25–4.28 (m, 4H, –OCH_2_CH_2_O–), 2.49 (d, *J* = 8.5 Hz, 2H), 2.02–2.07 (m, 2H), 1.93–1.95 (m, 1H), 1.73–1.81 (m, 3H), 1.47–1.62 (m, 5H), 1.29–1.34 (m, 4H), 1.19–1.22 (m, 4H), 1.16 (s, 3H), 1.05–1.12 (m, 4H), 0.97–1.01 (m, 1H), 0.92 (d, *J* = 6.5 Hz, 3H), 0.86 (d, *J* = 2.0 Hz, 3H), 0.84 (d, *J* = 2.0 Hz, 3H), 0.72 (s, 3H). HRMS (ESI): calculated for C_45_H_58_O_6_Na ([M + Na]^+^), 717.4134; found, 717.4131.

*Data for* ***Ic***: Yield: 32%, white solid, mp 106–108 °C; IR cm^−1^ (KBr): 2959, 2858, 1723, 1639, 1455, 1213, 679; ^1^H NMR (500 MHz, CDCl_3_) *δ*: 8.04 (d, *J* = 7.5 Hz, 2H, Ar-H), 7.69 (d, *J* = 16.0 Hz, 1H, H-3′), 7.52–7.55 (m, 3H, Ar-H), 7.41–7.44 (m, 2H, Ar-H), 7.37–7.38 (m, 3H, Ar-H), 6.44 (d, *J* = 16.0 Hz, 1H, H-2′), 5.36 (s, 1H, H-6), 5.22 (d, *J* = 8.5 Hz, 1H, H-7), 4.85–4.91 (m, 1H, H-3), 2.50 (d, *J* = 8.0 Hz, 2H), 2.03–2.07 (m, 2H), 1.93–1.97 (m, 1H), 1.73–1.83 (m, 3H), 1.47–1.60 (m, 5H), 1.29–1.33 (m, 4H), 1.20–1.24 (m, 4H), 1.17 (s, 3H), 1.04–1.14 (m, 4H), 0.97–1.01 (m, 1H), 0.92 (d, *J* = 6.5 Hz, 3H), 0.86 (d, *J* = 2.0 Hz, 3H), 0.84 (d, *J* = 2.5 Hz, 3H), 0.73 (s, 3H). HRMS (ESI): calculated for C_43_H_56_O_4_Na ([M + Na]^+^), 659.4030; found, 659.4076.

*Data for* ***Id***: Yield: 14%, white solid, mp 89–91 °C; IR cm^−1^ (KBr): 2955, 2860, 1713, 1640, 1510, 1457, 1276, 1231, 1168, 705; ^1^H NMR (500 MHz, CDCl_3_) *δ*: 8.04 (d, *J* = 7.5 Hz, 2H, Ar-H), 7.65 (d, *J* = 16.0 Hz, 1H, H-3′), 7.50–7.56 (m, 3H, Ar-H), 7.44 (t, *J* = 7.5 Hz, 2H, Ar-H), 7.05–7.08 (m, 2H, Ar-H), 6.36 (d, *J* = 16.0 Hz, 1H, H-2′), 5.35 (s, 1H, H-6), 5.22 (d, *J* = 9.0 Hz, 1H, H-7), 4.85–4.91 (m, 1H, H-3), 2.50 (d, *J* = 8.0 Hz, 2H), 2.03–2.06 (m, 2H), 1.93–1.97 (m, 1H), 1.73–1.82 (m, 3H), 1.47–1.60 (m, 5H), 1.29–1.33 (m, 4H), 1.19–1.22 (m, 4H), 1.17 (s, 3H), 1.06–1.12 (m, 4H), 0.97–0.99 (m, 1H), 0.93 (d, *J* = 6.5 Hz, 3H), 0.86 (d, *J* = 2.0 Hz, 3H), 0.84 (d, *J* = 2.0 Hz, 3H), 0.72 (s, 3H). HRMS (ESI): calculated for C_43_H_55_FO_4_Na ([M + Na]^+^), 677.3951; found, 677.3982.

*Data for* ***Ie***: Yield: 29%, white solid, mp 115–117 °C; IR cm^−1^ (KBr): 2944, 2862, 1711, 1635, 1507, 1459, 1278, 1167, 1116, 1069, 710; ^1^H NMR (500 MHz, CDCl_3_) *δ*: 8.02–8.04 (m, 2H, Ar-H), 7.63 (d, *J* = 16.0 Hz, 1H, H-3′), 7.52–7.56 (m, 1H, Ar-H), 7.41–7.46 (m, 4H, Ar-H), 7.34–7.36 (m, 2H, Ar-H), 6.40 (d, *J* = 16.0 Hz, 1H, H-2′), 5.35 (s, 1H, H-6), 5.21 (d, *J* = 8.5 Hz, 1H, H-7), 4.85–4.91 (m, 1H, H-3), 2.50 (d, *J* = 8.0 Hz, 2H), 2.02–2.07 (m, 2H), 1.93–1.97 (m, 1H), 1.73–1.84 (m, 3H), 1.41–1.63 (m, 5H), 1.27–1.34 (m, 4H), 1.19–1.24 (m, 4H), 1.16 (s, 3H), 1.06–1.13 (m, 4H), 0.97–1.01 (m, 1H), 0.92 (d, *J* = 6.5 Hz, 3H), 0.86 (d, *J* = 1.5 Hz, 3H), 0.84 (d, *J* = 2.0 Hz, 3H), 0.72 (s, 3H). HRMS (ESI): calculated for C_43_H_55_ClO_4_Na ([M + Na]^+^), 693.3681; found, 693.3687.

*Data for* ***If:*** Yield: 18%, white solid, mp 112–114 °C; IR cm^−1^ (KBr): 2945, 2860, 1711, 1638, 1456, 1273, 1171, 816, 706; ^1^H NMR (500 MHz, CDCl_3_) *δ*: 8.04 (d, *J* = 7.5 Hz, 2H, Ar-H), 7.61 (d, *J* = 16.0 Hz, 1H, H-3′), 7.50–7.56 (m, 3H, Ar-H), 7.41–7.44 (m, 2H, Ar-H), 7.37–7.39 (m, 2H, Ar-H), 6.42 (d, *J* = 16.0 Hz, 1H, H-2′), 5.35 (s, 1H, H-6), 5.21 (d, *J* = 8.5 Hz, 1H, H-7), 4.85–4.91 (m, 1H, H-3), 2.49 (d, *J* = 8.0 Hz, 2H), 2.03–2.06 (m, 2H), 1.93–1.96 (m, 1H), 1.70–1.84 (m, 3H), 1.45–1.62 (m, 5H), 1.30–1.35 (m, 4H), 1.20–1.24 (m, 4H), 1.16 (s, 3H), 1.06–1.13 (m, 4H), 0.97–1.01 (m, 1H), 0.92 (d, *J* = 4.0 Hz, 3H), 0.85 (s, 3H), 0.84 (s, 3H), 0.72 (s, 3H). HRMS (ESI): calculated for C_43_H_55_BrO_4_Na ([M + Na]^+^), 737.3172, 739.3169; found, 737.3181, 739.3161.

*Data for* ***Ig***: Yield: 20%, white solid, mp 98–100 °C; IR cm^−1^ (KBr): 2949, 2864, 1715, 1641, 1275, 1173, 1123, 1037, 1013, 985, 833, 709; ^1^H NMR (500 MHz, CDCl_3_) *δ*: 8.04 (d, *J* = 8.0 Hz, 2H, Ar-H), 7.69 (d, *J* = 16.0 Hz, 1H, H-3′), 7.63 (s, 4H, Ar-H), 7.53–7.56 (m, 1H, Ar-H), 7.41–7.44 (m, 2H, Ar-H), 6.51 (d, *J* = 16.0 Hz, 1H, H-2′), 5.35 (s, 1H, H-6), 5.23 (d, *J* = 8.5 Hz, 1H, H-7), 4.85–4.91 (m, 1H, H-3), 2.50 (d, *J* = 8.5 Hz, 2H), 2.04–2.06 (m, 2H), 1.94–1.97 (m, 1H), 1.73–1.83 (m, 3H), 1.45–1.62 (m, 5H), 1.30–1.36 (m, 4H), 1.22–1.25 (m, 4H), 1.17 (s, 3H), 1.07–1.12 (m, 4H), 0.98–1.01 (m, 1H), 0.93 (d, *J* = 6.0 Hz, 3H), 0.85 (s, 3H), 0.84 (s, 3H), 0.73 (s, 3H). HRMS (ESI): calculated for C_44_H_55_F_3_O_4_Na ([M + Na]^+^), 727.3950; found, 727.3958.

*Data for* ***Ih***: Yield: 53%, white solid, mp 90–92 °C; IR cm^−1^ (KBr): 2945, 2862, 1713, 1638, 1456, 1274, 1169, 808, 708; ^1^H NMR (500 MHz, CDCl_3_) *δ*: 8.04 (d, *J* = 7.5 Hz, 2H, Ar-H), 7.66 (d, *J* = 16.0 Hz, 1H, H-3′), 7.53–7.55 (m, 1H, Ar-H), 7.41–7.43 (m, 4H, Ar-H), 7.19 (d, *J* = 7.5 Hz, 2H, Ar-H), 6.39 (d, *J* = 16.5 Hz, 1H, H-2′), 5.36 (s, 1H, H-6), 5.21 (d, *J* = 8.5 Hz, 1H, H-7), 4.85–4.91 (m, 1H, H-3), 2.50 (d, *J* = 8.5 Hz, 2H), 2.37 (s, 3H), 2.03–2.05 (m, 2H), 1.93–1.96 (m, 1H), 1.73–1.82 (m, 3H), 1.46–1.64 (m, 5H), 1.30–1.36 (m, 4H), 1.22–1.25 (m, 4H), 1.17 (s, 3H), 1.06–1.12 (m, 4H), 0.97–1.01 (m, 1H), 0.92 (d, *J* = 4.5 Hz, 3H), 0.85 (s, 3H), 0.84 (s, 3H), 0.73 (s, 3H). HRMS (ESI): calculated for C_44_H_58_O_4_Na ([M + Na]^+^), 673.4227; found, 673.6233.

*Data for* ***Ii***: Yield: 30%, white solid, mp 100–102 °C; IR cm^−1^ (KBr): 2945, 2862, 1710, 1635, 1605, 1260, 1166, 824, 708; ^1^H NMR (500 MHz, CDCl_3_) *δ*: 8.04 (d, *J* = 7.0 Hz, 2H, Ar-H), 7.64 (d, *J* = 16.0 Hz, 1H, H-3′), 7.52–7.55 (m, 1H, Ar-H), 7.46–7.49 (m, 2H, Ar-H), 7.44 (t, *J* = 7.5 Hz, 2H, Ar-H), 6.88–6.91 (m, 2H, Ar-H), 6.30 (d, *J* = 15.5 Hz, 1H, H-2′), 5.36 (s, 1H, H-6), 5.21 (d, *J* = 8.5 Hz, 1H, H-7), 4.85–4.91 (m, 1H, H-3), 3.83 (s, 3H), 2.50 (d, *J* = 8.5 Hz, 2H), 2.03–2.05 (m, 2H), 1.93–1.96 (m, 1H), 1.72–1.84 (m, 3H), 1.46–1.60 (m, 5H), 1.30–1.38 (m, 4H), 1.22–1.25 (m, 4H), 1.16 (s, 3H), 1.06–1.12 (m, 4H), 0.97–1.01 (m, 1H), 0.92 (d, *J* = 3.5 Hz, 3H), 0.85 (s, 3H), 0.84 (s, 3H), 0.72 (s, 3H). HRMS (ESI): calculated for C_44_H_58_O_5_Na ([M + Na]^+^), 689.4197; found, 689.4182.

*Data for* ***IIa***: Yield: 16%, white solid, mp 98–100 °C; IR cm^−1^ (KBr): 2952, 2860, 1702, 1635, 1451, 1231, 1109, 808, 704; ^1^H NMR (500 MHz, CDCl_3_) *δ*: 7.91–8.04 (m, 2H, Ar-H), 7.59 (d, *J* = 16.0 Hz, 1H, H-3′), 7.44 (t, *J* = 7.5 Hz, 1H, Ar-H), 7.23 (d, *J* = 8.0 Hz, 1H, Ar-H), 7.00–7.03 (m, 2H, Ar-H), 6.81 (d, *J* = 7.5 Hz, 1H, Ar-H), 6.26 (d, *J* = 16.0 Hz, 1H, H-2′), 6.00 (s, 2H, –OCH_2_O–), 5.35 (s, 1H, H-6), 5.20 (d, *J* = 8.5 Hz, 1H, H-7), 4.83–4.91 (m, 1H, H-3), 2.47–2.50 (m, 2H), 2.40 (s, 2H), 2.03–2.05 (m, 2H), 1.92–1.95 (m, 1H), 1.71–1.83 (m, 3H), 1.59–1.62 (m, 2H), 1.47–1.54 (m, 3H), 1.30–1.36 (m, 4H), 1.18–1.24 (m, 5H), 1.16 (s, 3H), 1.06–1.12 (m, 4H), 0.97–1.01 (m, 1H), 0.92 (d, *J* = 6.0 Hz, 3H), 0.85 (s, 3H), 0.84 (s, 3H), 0.72 (s, 3H). HRMS (ESI): calculated for C_45_H_58_O_6_Na ([M + Na]^+^), 717.4131; found, 717.4138.

*Data for* ***IIb:*** Yield: 21%, white solid, mp 107–109 °C; IR cm^−1^ (KBr): 2941, 2861, 1711, 1638, 1509, 1461, 1280, 805, 748; ^1^H NMR (500 MHz, CDCl_3_) *δ*: 7.92 (d, *J* = 8.0 Hz, 2H, Ar-H), 7.57 (d, *J* = 16.0 Hz, 1H, H-3´), 7.23 (d, *J* = 8.0 Hz, 2H, Ar-H), 7.02–7.05 (m, 2H, Ar-H), 6.86 (d, *J* = 8.5 Hz, 1H, Ar-H), 6.28 (d, *J* = 16.0 Hz, 1H, H-2´), 5.35 (s, 1H, H-6), 5.19 (d, *J* = 8.5 Hz, 1H, H-7), 4.83–4.89 (m, 1H, H-3), 4.26–4.29 (m, 4H, –OCH_2_CH_2_O–), 2.48 (d, *J* = 8.5 Hz, 2H), 2.40 (s, 3H), 2.01–2.05 (m, 2H), 1.91–1.95 (m, 1H), 1.74–1.79 (m, 3H), 1.44–1.54 (m, 5H), 1.29–1.31 (m, 3H), 1.20–1.22 (m, 5H), 1.16 (s, 3H), 1.05–1.09 (m, 4H), 0.99–1.00 (m, 1H), 0.92 (d, *J* = 6.5 Hz, 3H), 0.86 (d, *J* = 2.0 Hz, 3H), 0.84 (d, *J* = 2.5 Hz, 3H), 0.72 (s, 3H). HRMS (ESI): calculated for C_46_H_60_O_6_Na ([M + Na]^+^), 731.4288; found, 731.4285.

*Data for* ***IIc:*** Yield: 45%, white solid, mp 112–114 °C; IR cm^−1^ (KBr): 2940, 2862, 1706, 1640, 1273, 1175, 1112, 990, 944, 758; ^1^H NMR (500 MHz, CDCl_3_) *δ*: 7.93 (d, *J* = 8.0 Hz, 2H, Ar-H), 7.69 (d, *J* = 16.0 Hz, 1H, H-3′), 7.51–7.53 (m, 2H, Ar-H), 7.36–7.38 (m, 3H, Ar-H), 7.22 (d, *J* = 8.0 Hz, 2H, Ar-H), 6.44 (d, *J* = 16.0 Hz, 1H, H-2′), 5.36 (s, 1H, H-6), 5.22 (d, *J* = 8.5 Hz, 1H, H-7), 4.83–4.90 (m, 1H, H-3), 2.49 (d, *J* = 8.0 Hz, 2H), 2.39 (s, 3H), 2.03–2.05 (m, 2H), 1.92–1.95 (m, 1H), 1.72–1.85 (m, 3H), 1.46–1.61 (m, 4H), 1.30–1.38 (m, 4H), 1.19–1.26 (m, 5H), 1.16 (s, 3H), 1.03–1.13 (m, 4H), 0.97–1.01 (m, 1H), 0.92 (d, *J* = 6.5 Hz, 3H), 0.86 (d, *J* = 2.0 Hz, 3H), 0.84 (d, *J* = 2.0 Hz, 3H), 0.72 (s, 3H). HRMS [ESI]: calculated for C_44_H_58_O_4_Na ([M + Na]^+^), 673.4233; found, 673.4231.

*Data for* ***IId:*** Yield: 19%, white solid, mp 108–110 °C; IR cm^−1^ (KBr): 2948, 2863, 1712, 1639, 1609, 1274, 1168, 1108, 829, 748; ^1^H NMR (500 MHz, CDCl_3_) *δ*: 7.92 (d, *J* = 7.5 Hz, 2H, Ar-H), 7.64 (d, *J* = 16.0 Hz, 1H, H-3′), 7.50–7.52 (m, 2H, Ar-H), 7.23 (d, *J* = 8.5 Hz, 2H, Ar-H), 7.05–7.08 (m, 2H, Ar-H), 6.36 (d, *J* = 16.5 Hz, 1H, H-2′), 5.34 (s, 1H, H-6), 5.21 (d, *J* = 8.5 Hz, 1H, H-7), 4.83–4.89 (m, 1H, H-3), 2.49 (d, *J* = 8.0 Hz, 2H), 2.40 (s, 3H), 2.03–2.05 (m, 2H), 1.93–1.95 (m, 1H), 1.69–1.82 (m, 3H), 1.57–1.63 (m, 2H), 1.45–1.50 (m, 3H), 1.31–1.36 (m, 4H), 1.21–1.25 (m, 4H), 1.16 (s, 3H), 1.06–1.12 (m, 4H), 0.97–1.01 (m, 1H), 0.92 (d, *J* = 5.0 Hz, 3H), 0.85 (s, 3H), 0.84 (s, 3H), 0.72 (s, 3H). HRMS (ESI): calculated for C_44_H_57_FO_4_Na ([M + Na]^+^), 691.4139; found, 691.4138.

*Data for* ***IIe:*** Yield: 27%, white solid, mp 96–98 °C; IR cm^−1^ (KBr): 2927, 2854, 1727, 1643, 1215, 1113, 806, 690; ^1^H NMR (500 MHz, CDCl_3_) *δ*: 7.92 (d, *J* = 8.0 Hz, 2H, Ar-H), 7.63 (d, *J* = 16.0 Hz, 1H, H-3′), 7.44–7.46 (m, 2H, Ar-H), 7.33–7.36 (m, 2H, Ar-H), 7.23 (d, *J* = 8.0 Hz, 2H, Ar-H), 6.40 (d, *J* = 16.0 Hz, 1H, H-2′), 5.34 (s, 1H, H-6), 5.21 (d, *J* = 8.5 Hz, 1H, H-7), 4.83–4.89 (m, 1H, H-3), 2.48 (d, *J* = 8.5 Hz, 2H), 2.40 (s, 3H), 2.03–2.06 (m, 2H), 1.92–1.95 (m, 1H), 1.74–1.81 (m, 3H), 1.44–1.55 (m, 5H), 1.29–1.31 (m, 3H), 1.19–1.23 (m, 5H), 1.16 (s, 3H), 1.06–1.10 (m, 4H), 0.97–0.99 (m, 1H), 0.92 (d, *J* = 6.5 Hz, 3H), 0.86 (d, *J* = 2.5 Hz, 3H), 0.84 (d, *J* = 2.0 Hz, 3H), 0.72 (s, 3H). HRMS (ESI): calculated for C_44_H_57_ClO_4_Na ([M + Na]^+^), 707.3843; found, 707.3843.

*Data for* ***IIf:*** Yield: 20%, white solid, mp 96–98 °C; IR cm^−1^ (KBr): 2946, 2862, 1713, 1637, 1272, 1171, 1110, 818, 748; ^1^H NMR (500 MHz, CDCl_3_) *δ*: 7.91–8.04 (m, 2H, Ar-H), 7.61 (d, *J* = 16.0 Hz, 1H, H-3′), 7.52 (d, *J* = 7.0 Hz, 2H, Ar-H), 7.39 (d, *J* = 7.0 Hz, 2H, Ar-H), 7.23 (d, *J* = 7.0 Hz, 2H, Ar-H), 6.42 (d, *J* = 16.0 Hz, 1H, H-2′), 5.34 (s, 1H, H-6), 5.21 (d, *J* = 8.5 Hz, 1H, H-7), 4.84–4.90 (m, 1H, H-3), 2.48 (d, *J* = 8.5 Hz, 2H), 2.40 (s, 2H), 2.03–2.06 (m, 2H), 1.93–1.95 (m, 1H), 1.72–1.84 (m, 3H), 1.59–1.63 (m, 2H), 1.44–1.52 (m, 3H), 1.30–1.37 (m, 4H), 1.20–1.26 (m, 5H), 1.16 (s, 3H), 1.04–1.12 (m, 4H), 0.97–1.01 (m, 1H), 0.92 (d, *J* = 4.5 Hz, 3H), 0.85 (s, 3H), 0.84 (s, 3H), 0.72 (s, 3H). HRMS (ESI): calculated for C_44_H_57_BrO_4_Na ([M + Na]^+^), 751.3338; found, 751.3333.

*Data for* ***IIg:*** Yield: 18%, white solid, mp 84–86 °C; IR cm^−1^ (KBr): 2948, 2864, 1715, 1275, 1173, 1122, 833, 748; ^1^H NMR (500 MHz, CDCl_3_) *δ*: 7.92 (d, *J* = 7.5 Hz, 2H, Ar-H), 7.69 (d, *J* = 16.0 Hz, 1H, H-3′), 7.63 (s, 4H, Ar-H), 7.23 (d, *J* = 8.0 Hz, 2H, Ar-H), 6.50 (d, *J* = 16.0 Hz, 1H, H-2′), 5.35 (s, 1H, H-6), 5.23 (d, *J* = 8.0 Hz, 1H, H-7), 4.82–4.90 (m, 1H, H-3), 2.49 (d, *J* = 8.0 Hz, 2H), 2.40 (s, 3H), 2.03–2.06 (m, 2H), 1.93–1.96 (m, 1H), 1.67–1.85 (m, 4H), 1.47–1.62 (m, 4H), 1.30–1.41 (m, 4H), 1.21–1.23 (m, 4H), 1.17 (s, 3H), 1.06–1.12 (m, 4H), 0.96–1.01 (m, 1H), 0.93 (d, *J* = 6.0 Hz, 3H), 0.85 (s, 3H), 0.84 (s, 3H), 0.73 (s, 3H). HRMS (ESI): calculated for C_45_H_57_F_3_O_4_Na ([M + Na]^+^), 741.4107; found, 741.4101.

*Data for* ***IIh:*** Yield: 26%, white solid, mp 93–95 °C; IR cm^−1^ (KBr): 2947, 2863, 1712, 1635, 1272, 1169, 1109, 811, 749; ^1^H NMR (500 MHz, CDCl_3_) *δ*: 7.92 (d, *J* = 7.5 Hz, 2H, Ar-H), 7.66 (d, *J* = 16.0 Hz, 1H, H-3′), 7.43 (d, *J* = 7.5 Hz, 2H, Ar-H), 7.23 (d, *J* = 7.5 Hz, 2H, Ar-H), 7.19 (d, *J* = 7.5 Hz, 2H, Ar-H), 6.39 (d, *J* = 16.0 Hz, 1H, H-2′), 5.35 (s, 1H, H-6), 5.21 (d, *J* = 8.5 Hz, 1H, H-7), 4.82–4.89 (m, 1H, H-3), 2.49 (d, *J* = 8.5 Hz, 2H), 2.40 (s, 3H), 2.37 (s, 3H), 2.03–2.05 (m, 2H), 1.92–1.95 (m, 1H), 1.71–1.84 (m, 3H), 1.60 (s, 2H), 1.47–1.54 (m, 3H), 1.30–1.41 (m, 4H), 1.19–1.23 (m, 4H), 1.16 (s, 3H), 1.03–1.12 (m, 4H), 0.97–1.01 (m, 1H), 0.92 (d, *J* = 4.5 Hz, 3H), 0.85 (s, 3H), 0.84 (s, 3H), 0.72 (s, 3H). HRMS (ESI): calculated for C_45_H_60_O_4_Na ([M + Na]^+^), 687.4389; found, 687.4390.

*Data for* ***IIi:*** Yield: 34%, white solid, mp 97–99 °C; IR cm^−1^ (KBr): 2946, 2863, 1711, 1634, 1607, 1263, 1167, 826, 749; ^1^H NMR (500 MHz, CDCl_3_) *δ*: 7.91–8.04 (m, 2H, Ar-H), 7.64 (d, *J* = 16.0 Hz, 1H, H-3′), 7.48 (d, *J* = 7.5 Hz, 2H, Ar-H), 7.23 (d, *J* = 7.5 Hz, 2H, Ar-H), 6.90 (d, *J* = 8.0 Hz, 2H, Ar-H), 6.31 (d, *J* = 16.0 Hz, 1H, H-2′), 5.35 (s, 1H, H-6), 5.21 (d, *J* = 8.5 Hz, 1H, H-7), 4.83–4.90 (m, 1H, H-3), 3.83 (s, 3H), 2.47–2.50 (m, 2H), 2.40 (s, 3H), 2.03–2.05 (m, 2H), 1.92–1.95 (m, 1H), 1.69–1.82 (m, 3H), 1.61 (s, 2H), 1.46–1.54 (m, 3H), 1.31–1.37 (m, 4H), 1.20–1.23 (m, 4H), 1.16 (s, 3H), 1.03–1.13 (m, 4H), 0.97–1.01 (m, 1H), 0.92 (d, *J* = 5.0 Hz, 3H), 0.85 (s, 3H), 0.84 (s, 3H), 0.72 (s, 3H). HRMS (ESI): calculated for C_45_H_60_O_5_Na ([M + Na]^+^), 703.4338; found, 703.4337.

### 2.5. Biological Assay

#### 2.5.1. Insecticidal Activity of Compounds **I**(**a**–**i**)–**II**(**a**–**i**) against *M. separata*

Thirty-six early 3rd-instar larvae of *M. separata* were selected as the tested insects for each compound. Solutions of tested compounds (rotenone as a positive control) were prepared in acetone (containing 0.5% *N*-methylpyrrolidone) at 1 mg/mL. After being dipped into the corresponding solution for 3 s, wheat leaf discs (1 × 1 cm) were taken out and dried. Wheat leaf discs were treated by acetone (containing 0.5% *N*-methylpyrrolidone) alone as the blank control group (CK). Some of the above discs were added to each culture dish (12 insects per dish). Once the discs were consumed, additional ones were added. After 48 h, the rest of compound-soaked discs were cleaned out, and the untreated ones were added until the end of pupae (temperature: 25 ± 2 °C; RH: 65–80%; photoperiod: light/dark = 12 h/12 h). Their corrected final mortality rate values were calculated as follows: corrected mortality rate (%) = (*T* − *C*) × 100/(100% − *C*); *C* is the mortality rate of CK, and *T* is the mortality rate of the treated *M. separata* [4,24].

#### 2.5.2. Insecticidal Activity of Compounds **I**(**a**–**i**)–**II**(**a**–**i**) against *P. xylostella*

For each compound, 45 3rd-instar larvae of *P. xylostella* were selected. Solutions of tested compounds (*β*-cypermethrin as a positive control) were prepared in acetone (containing 0.5% *N*-methylpyrrolidone) at 1 mg/mL. Fresh cabbage leaf discs (0.5 × 0.5 cm) were dipped into the corresponding solution for 3 s and dried. Fresh cabbage leaf discs (0.5 × 0.5 cm) were treated by acetone (containing 0.5% *N*-methylpyrrolidone) alone as the blank control group (CK). Some of the above discs were added to each culture dish (15 insects per dish). Once the discs were consumed, additional ones were added (temperature: 25 ± 2 °C; RH: 65–80%; photoperiod: light/dark = 14 h/10 h). Their 24 and 48 h corrected mortality rate values were calculated in the same way as mentioned above. Finally, the 48 h LC_50_ values of some potent compounds were calculated [24].

## 3. Results and Discussion

### 3.1. Chemistry

**Scheme 1:**
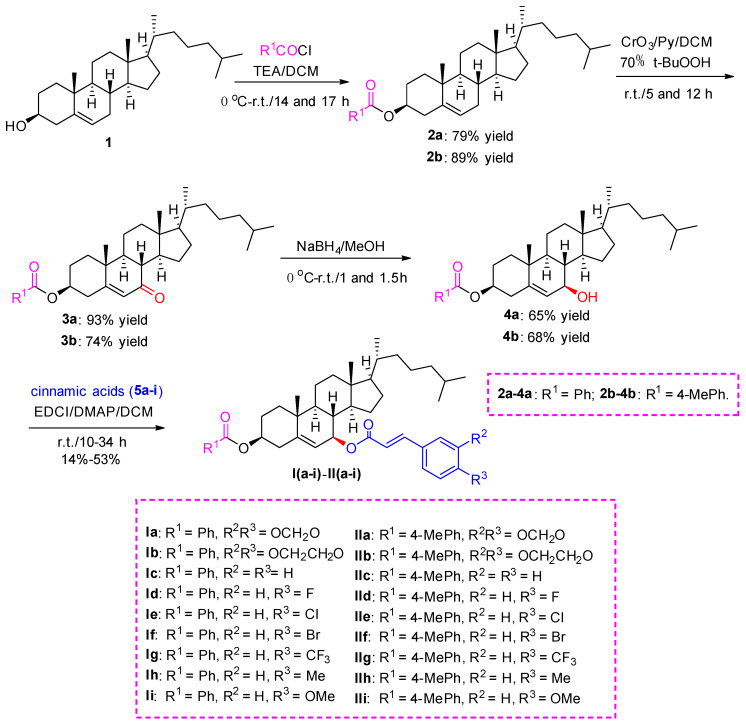
Synthesis of target compounds **I**(**a–i**)−**II**(**a–i**).

**Figure 2:**
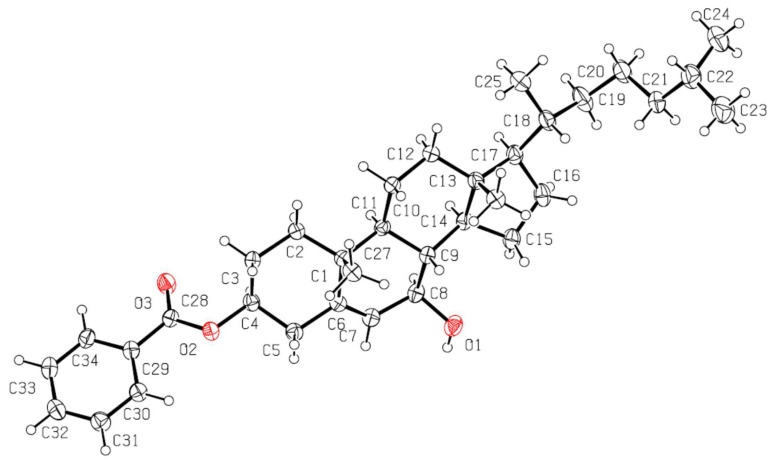
X-ray crystal structure of compound **4a**.

As shown in Scheme 1, intermediates **2a** and **2b** were prepared in 79 and 89% yields from one reacting with acyl chlorides [4,24]. Then, compounds **2a** and **2b** were oxidized by CrO_3_/*t*-BuOOH to give compounds **3a** and **3b** in 93% and 74% yields, respectively. In the presence of sodium borohydride, to our delight, the carbonyl group at the C-7 position of **3a** and **3b** was stereoselectively reduced to β-OH, and the corresponding compounds **4a** and **4b** were obtained in 65% and 68% yields, respectively. Finally, compounds **4a** and **4b** reacted with the cinnamic acid-like fragments (**5a**–**i**) to afford target compounds **I**(**a**–**i**)−**II**(**a**–**i**) in 14–53% yields [7]. Their structures were characterized by HRMS, melting points, IR, and ^1^H NMR. Notably, the steric structure of **4a** (CCDC: 2214110) was further determined by X-ray diffraction (Figure 2). Obviously, configurations of the ester group at the C-3 position and the hydroxyl group at the C-7 position of **4a** were all β.

### 3.2. Insecticidal Activities

The results of the insecticidal activity of **I**(**a**–**i**)–**II**(**a**–**i**) against *M. separata* treated at 1 mg/mL are shown in Table 1. Among them, the corrected final mortality rates (CFMRs) of **2a**, **Id**, **Ig**, and **IIg** were 45.4%, 48.4%, 51.5%, and 46.8%, respectively, whereas the CFMR of the precursor **1** was only 21.2%; that is, the growth-inhibitory activity of compounds **2a**, **Id**, **Ig**, and **IIg** was 2.1–2.4-fold that of cholesterol. Compared with **1**, compounds **2a**,**b**; **3a**,**b**; and **4a**,**b** showed better insecticidal activity, indicating that structural modifications at the C-3 and C-7 positions were necessary for insecticidal activity. Moreover, compounds **2a**–**4a** (R^1^ = Ph) showed more promising insecticidal activity than the corresponding ones **2b**–**4b** (R^1^ = 4-MePh) (e.g., **2a** vs. **2b**; **3a** vs. **3b**; **4a** vs. **4b**). To compounds **Ia**–**Ii** (R^1^ = Ph), the introduction of cinnamic acids containing the fluorine atom or CF_3_ on the hydroxyl group of compound **4a** resulted in promising compounds. For example, the CFMRs of **Id** (R^2^ = H, R^3^ = 4-F) and **Ig** (R^2^ = H, R^3^ = 4-CF_3_) were 48.4% and 51.5%, respectively, and the CFMRs of compounds **Ie** (R^2^ = H, R^3^ = 4-Cl) and **If** (R^2^ = H, R^3^ = 4-Br) were the same as that of **4a**. Although the CFMR of **Ii** (R^2^ = H, R^3^ = 4-OMe) was 42.4%, the CFMRs of **Ia** (R^2^R^3^ = 3,4-OCH_2_O-), **Ib** (R^2^R^3^ = 3,4-OCH_2_CH_2_O-), and **Ih** (R^2^ = H, R^3^ = 4-Me) were only 33.3%, 36.3%, and 30.3%, respectively. The same structure–activity relationships were also observed for compounds **IIa**–**IIi** (R^1^ = 4-MePh): for instance, the CFMRs of **IId** (R^2^ = H, R^3^ = 4-F) and **IIg** (R^2^ = H, R^3^ = 4-CF_3_) were 37.5% and 46.8%, respectively, whereas the CFMRs of **IIa**–**c**,**e**,**f**,**h**,**i** were 25.0–34.3%. This demonstrates that the introduction of the fluorine atom or CF_3_ on the para position of the phenyl of **Ic** and **IIc** was very important for the insecticidal activity against *M. separata.*

The results of the insecticidal activity of **I**(**a**–**i**)–**II**(**a–i**) against *P. xylostella* treated at 1 mg/mL are shown in Table 2. Among them, the CFMRs of **Ig**, **IIf**, and **IIi** were 40.9%, 44.1%, and 41.8%, respectively, whereas the CFMR of the precursor **1** was only 18.6%. Similarly, compounds **2a**–**4a** (R^1^ = Ph) showed better insecticidal activity against *P. xylostella* than the corresponding ones **2b**–**4b** (R^1^ = 4-MePh); for compounds **Ia**–**Ii** (R^1^ = Ph), the introduction of cinnamic acids containing the fluorine atom or CF_3_ on the hydroxyl group of compound **4a** can result in the promising compounds **Id** (R^2^ = H, R^3^ = 4-F) and **Ig** (R^2^ = H, R^3^ = 4-CF_3_). However, for **IIa**–**IIi** (R^1^ = 4-MePh), compounds **IIf** (R^2^ = H, R^3^ = 4-Br) and **IIi** (R^2^ = H, R^3^ = 4-OMe) showed potent insecticidal activity against *P. xylostella.*

As described in Table 3, three potent compounds, **Ig**, **IIf**, and **IIi**, were further evaluated against *P. xylostella*, and their LC_50_ values were 1.560, 1.390, and 1.508 mg/mL, respectively. Namely, compared with that of their precursor **1** (LC_50_: 2.946 mg/mL), they exhibited 1.9–2.1-fold more potent insecticidal activity.

## 4. Conclusions

In summary, to develop new natural product-based pesticide candidates, a series of new cholesterol ester derivatives containing cinnamic acid-like fragments at the C-7 position were prepared. Against *M. separata*, compounds **2a**, **Id**, **Ig**, and **IIg** showed 2.1–2.4 times more potent growth-inhibitory activity than cholesterol. Against *P. xylostella*, compounds **Ig**, **IIf**, and **IIi** exhibited 1.9–2.1 times more promising insecticidal activity than cholesterol. The structure–activity relationships of the compounds suggest that the introduction of the fluorine atom or CF_3_ on the para position of the phenyl of **Ic** and **IIc** was very important for the insecticidal activity against *M. separata*; and against *P. xylostella*, the introduction of the fluorine atom or CF_3_ on the para position of the phenyl of **Ic** was necessary for insecticidal activity, whereas the introduction of the bromine atom or methoxy on the para position of the phenyl of **IIc** was vital for insecticidal activity. These results will provide a basis for guiding the synthesis of novel cholesterol derivatives as new pesticidal agents in the future.

## Figures and Tables

**Table 1:** Insecticidal activity of compounds **I**(**a**–**i**)–**II**(**a**–**i**) against *M. separata* at 1 mg/mL.

Compound	Corrected Final Mortality Rate (Mean ± SE, %) ^a^
**1**	21.2 ± 2.7
**2a**	45.4 ± 4.8
**2b**	28.1 ± 2.7
**3a**	42.4 ± 2.7
**3b**	31.2 ± 2.7
**4a**	39.3 ± 2.7
**4b**	34.3 ± 4.8
**Ia**	33.3 ± 2.7
**Ib**	36.3 ± 4.8
**Ic**	27.2 ± 4.8
**Id**	48.4 ± 2.7
**Ie**	39.3 ± 2.7
**If**	39.3 ± 2.7
**Ig**	51.5 ± 2.7
**Ih**	30.3 ± 2.7
**Ii**	42.4 ± 2.7
**IIa**	25.0 ± 4.8
**IIb**	31.2 ± 2.7
**IIc**	28.1 ± 2.7
**IId**	37.5 ± 5.5
**IIe**	25.0 ± 4.8
**IIf**	34.3 ± 4.8
**IIg**	46.8 ± 2.7
**IIh**	31.2 ± 2.7
**IIi**	28.1 ± 2.7
rotenone	87.8 ± 2.7

^a^ Values are mean ± SE of three replicates.

**Table 2:** The insecticidal activity of compounds **I**(**a**–**i**)–**II**(**a**–**i**) against *P. xyllostella* at 1 mg/mL.

Compound	Corrected Mortality Rate (Mean ± SE, %) ^a^	
	24 h	48 h
**1**	6.8 ± 2.2	18.6 ± 2.2
**2a**	13.6 ± 2.2	23.2 ± 0
**2b**	15.5 ± 2.2	20.4 ± 2.2
**3a**	15.9 ± 2.2	25.5 ± 2.2
**3b**	11.1 ± 2.2	22.7 ± 2.2
**4a**	22.2 ± 2.2	29.5 ± 2.2
**4b**	17.7 ± 2.2	25.0 ± 3.8
**Ia**	22.2 ± 2.2	31.8 ± 3.8
**Ib**	11.1 ± 2.2	25.0 ± 3.8
**Ic**	13.3 ± 0	22.7 ± 2.2
**Id**	24.4 ± 2.2	38.6 ± 3.8
**Ie**	8.9 ± 2.2	29.5 ± 2.2
**If**	20.0 ± 0	36.3 ± 2.2
**Ig**	26.6 ± 3.8	40.9 ± 2.2
**Ih**	15.5 ± 2.2	27.2 ± 2.2
**Ii**	17.7 ± 2.2	34.0 ± 2.2
**IIa**	15.9 ± 2.2	27.9 ± 2.2
**IIb**	20.0 ± 3.8	36.3 ± 2.2
**IIc**	13.3 ± 3.8	20.4 ± 2.2
**IId**	15.9 ± 2.2	25.5 ± 2.2
**IIe**	22.2 ± 2.2	34.0 ± 2.2
**IIf**	25.0 ± 3.8	44.1 ± 3.8
**IIg**	20.4 ± 2.2	34.8 ± 4.4
**IIh**	11.3 ± 0	18.6 ± 2.2
**IIi**	20.4 ± 2.2	41.8 ± 2.2
*β*-cypermethrin	56.8 ± 2.2	97.6 ± 2.2

^a^ Values are mean ± SE of three replicates.

**Table 3:** LC_50_ values at 48 h of compounds **1**, **Ig**, **IIf**, and **IIi** against *P. xyllostella* ^a^.

Compound	Linear Regression Equation	LC_50_ (mg/mL)	Confidence Interval 95% (mg/mL)	*r*
**1**	Y = −0.816 + 1.738 X	2.946	2.293–3.923	0.992
**Ig**	Y = −0.253 + 1.308 X	1.560	1.136–2.329	0.989
**IIf**	Y = –0.201 + 1.405 X	1.390	1.021–1.982	0.997
**IIi**	Y = −0.239 + 1.339 X	1.508	1.095–2.232	0.998
*β*-cypermethrin	Y = 1.583 + 2.054 X	0.170	0.132–0.211	0.989

^a^ Values are mean ± SE of three replicates.

## Data Availability

Not applicable.

## Supplementary Materials

The following are available online at https://www.mdpi.com/article/10.3390/molecules27238437/s1, ^1^H NMR spectra.

## Sample Availability

Samples of the compounds are available from the authors.
