# Supplementary material for: Semisynthesis and Pesticidal Activities of Novel Cholesterol Ester Derivatives Containing Cinnamic Acid-like Fragments
Source: Molecules. 2022 Dec 2;27(23):8437. doi: 10.3390/molecules27238437 (PMC9735938; doi:10.3390/molecules27238437)

Supporting Information

Semisynthesis and Pesticidal Activities of Novel Cholesterol Esters Derivatives Containing Cinnamic Acids-Like Fragments

Rongfei Lu,[1,#] Jianwei Xu,[1,#] Hailong Wang,[3] Zhen Wang,[1] Shaoyong Zhang,[2] Hui Xu,[1,*] and Min Lv[1,*]

[1]College of Plant Protection, Northwest A&F University, Yangling 712100, Shaanxi Province, P.R. China

[2]Key Laboratory of Vector Biology and Pathogen Control of Zhejiang Province, College of Life Science, Huzhou University, Huzhou 313000, Zhejiang Province, P.R. China

[3]Yan'an Institute of Agricultural Sciences, Yan'an 716000, Shaanxi Province, P.R. China

*Correspondence: orgxuhui@nwsuaf.edu.cn; lvmin@nwsuaf.edu.cn

#These authors contributed equally to this work.

^{1}H NMR spectra

compound ***2a***

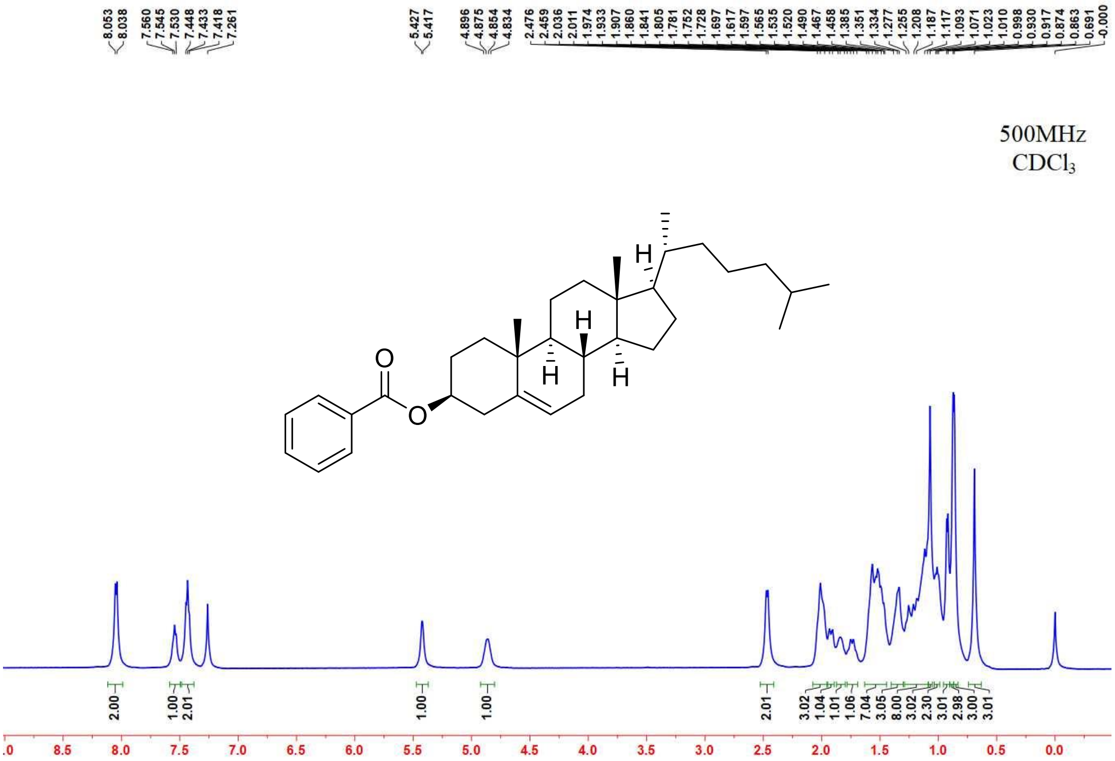

compound ***2b***

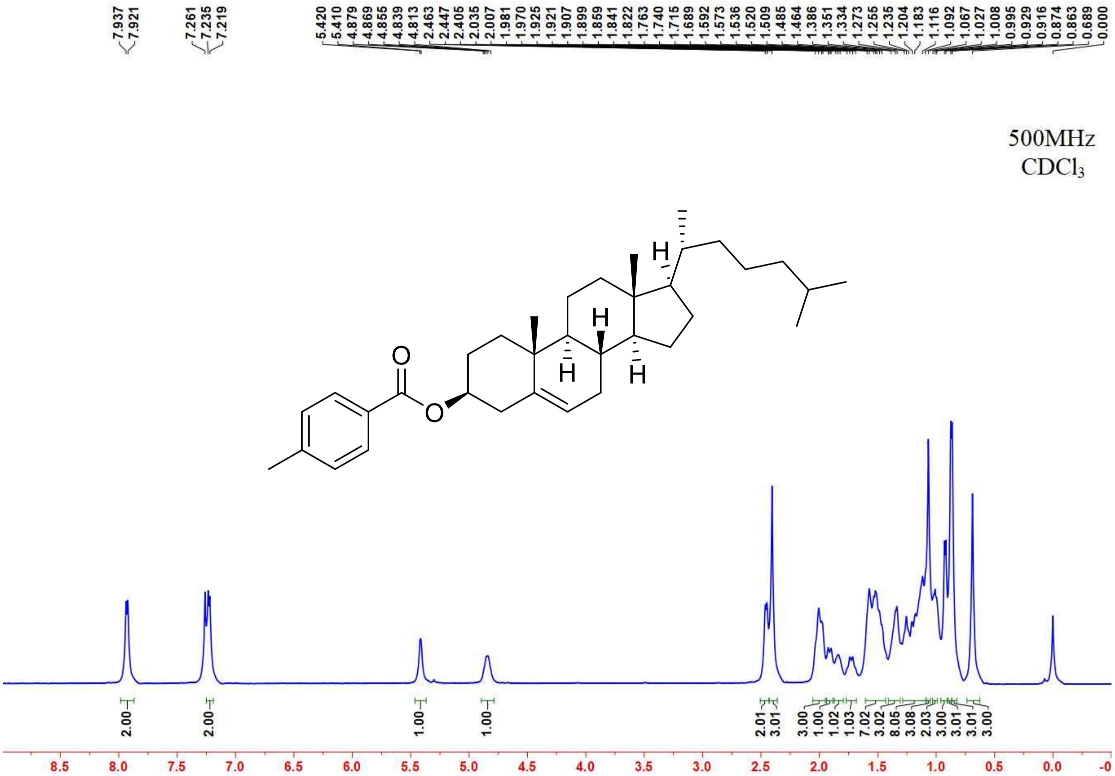

compound ***3a***

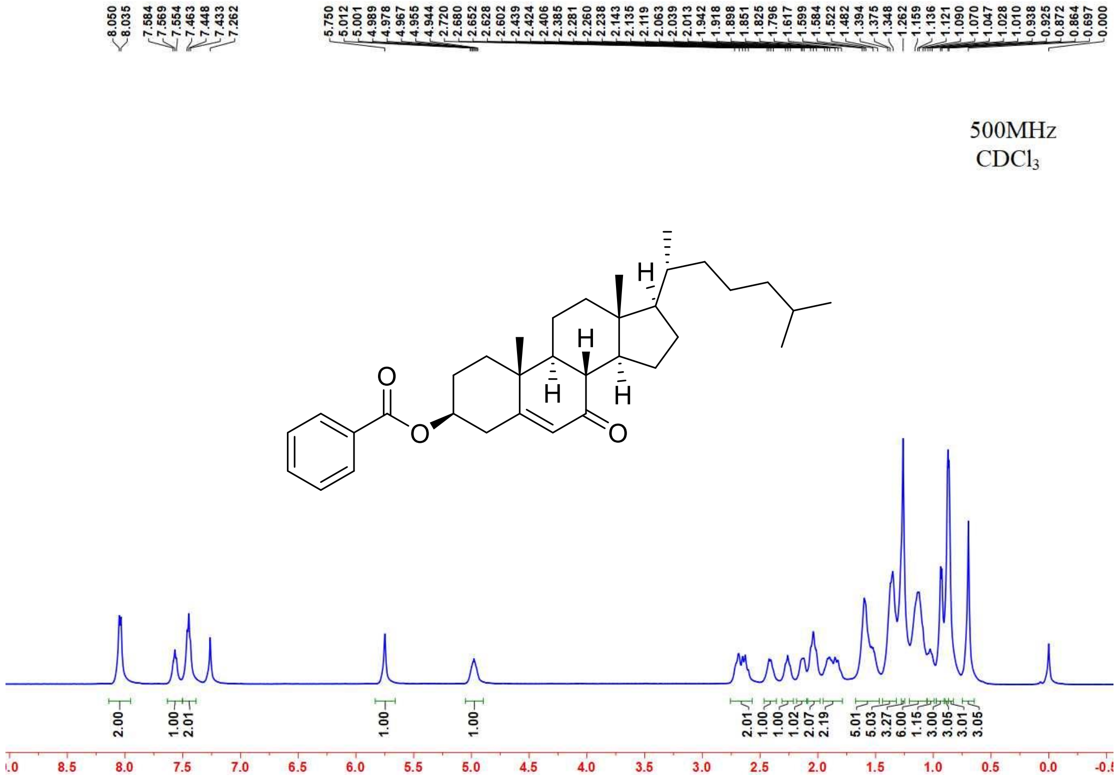

compound ***3b***

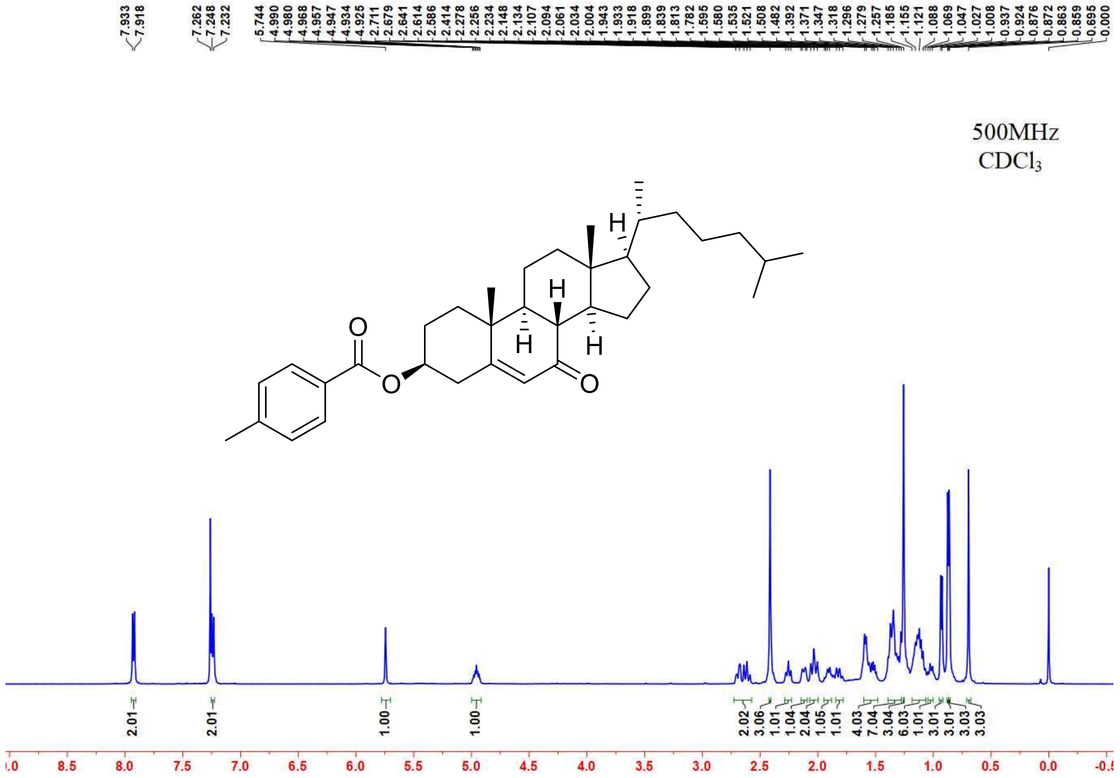

compound ***4a***

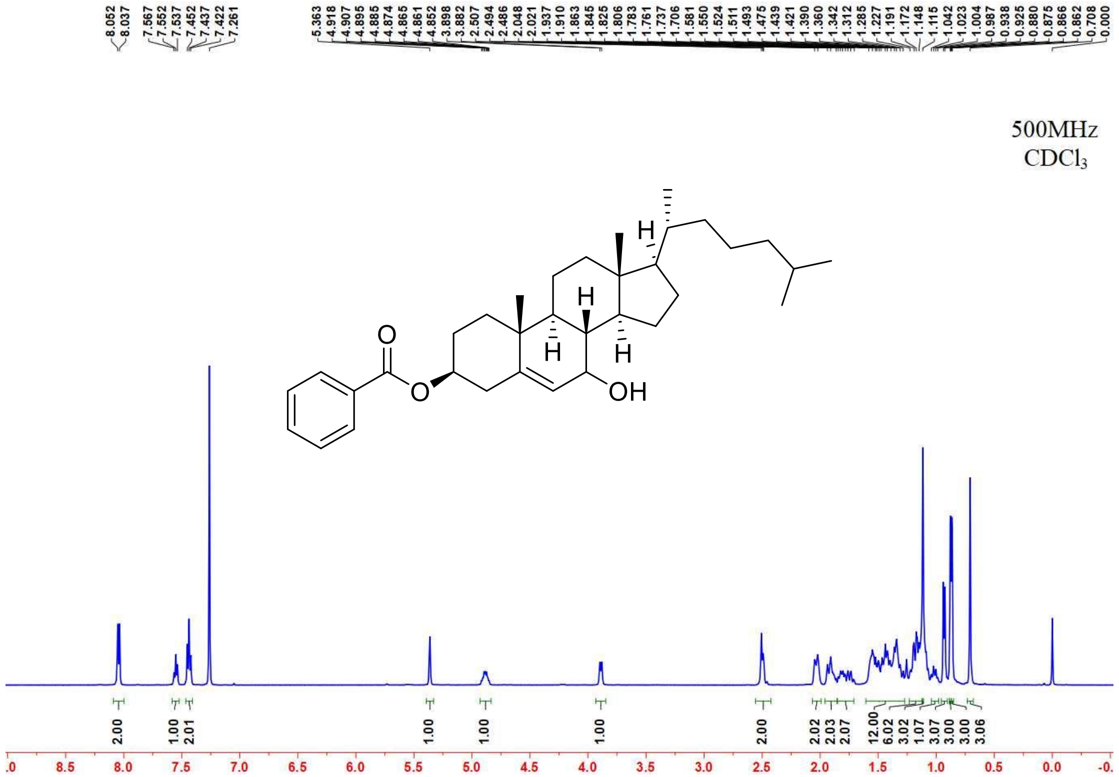

compound ***4b***

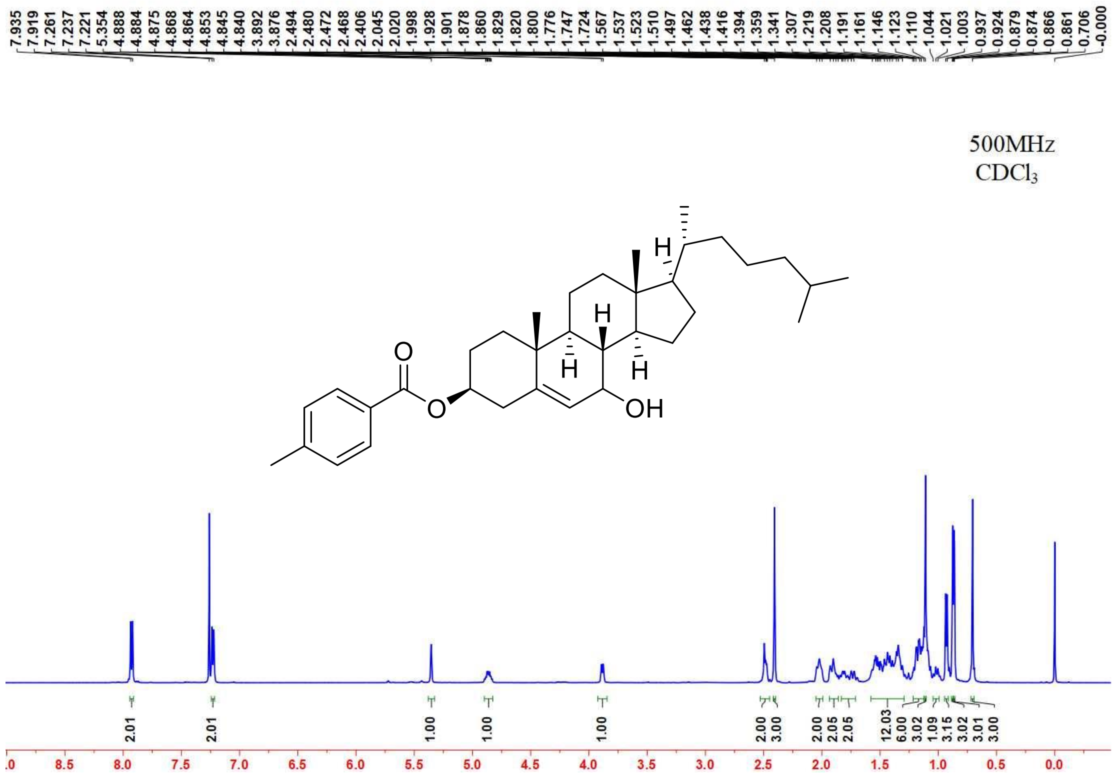

compound ***Ia***

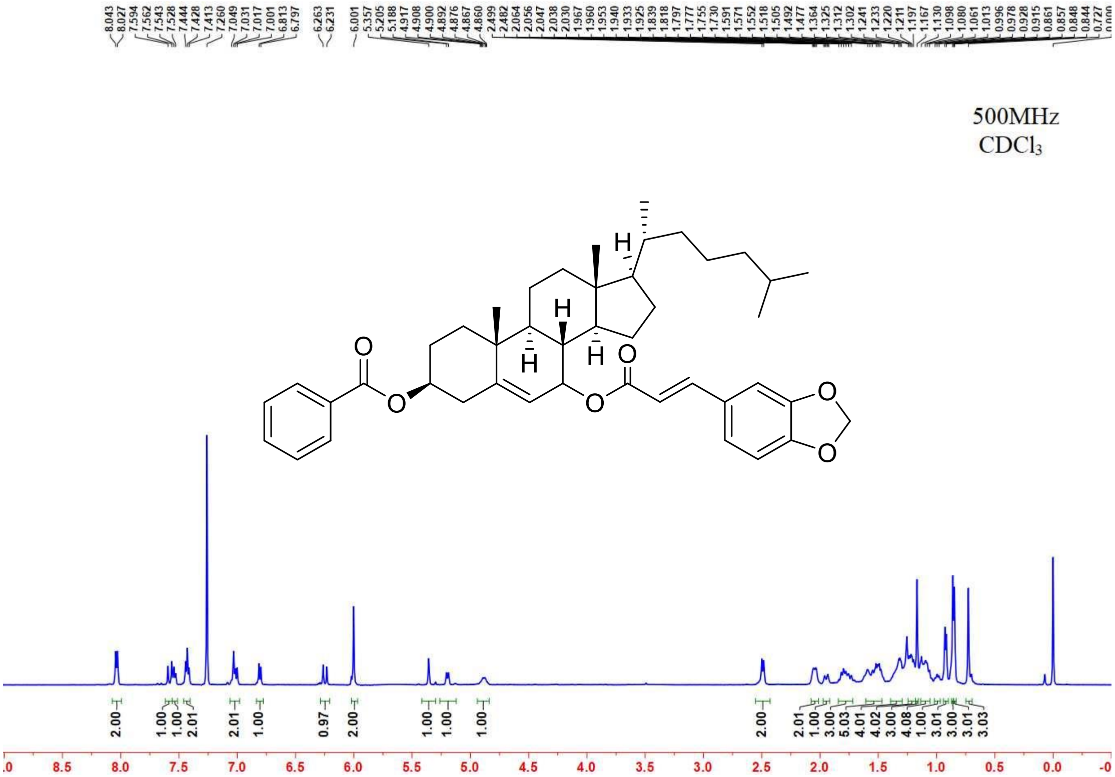

compound ***Ib***

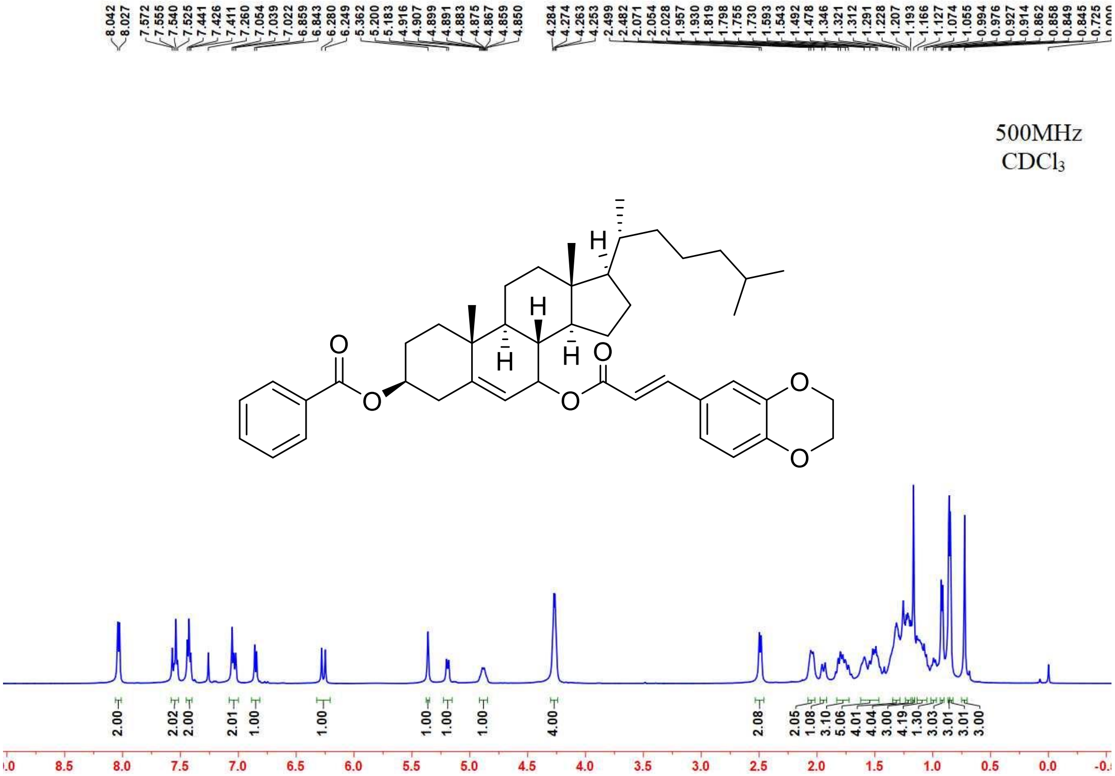

compound ***Ic***

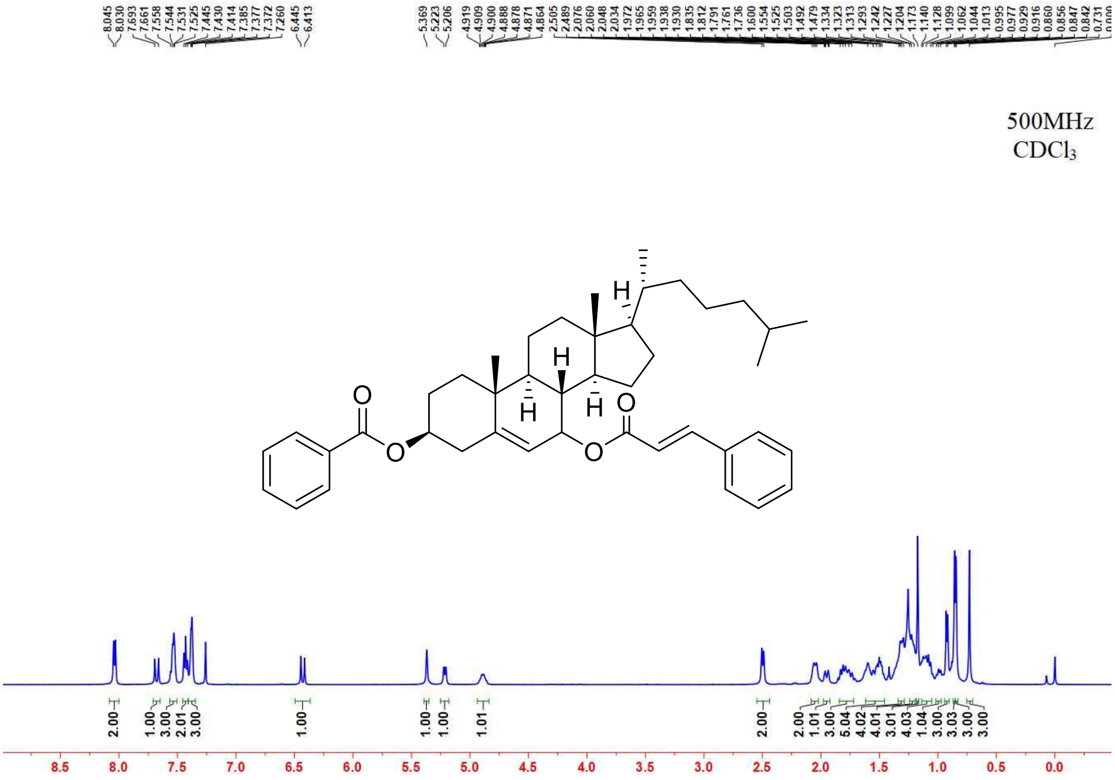

compound ***Id***

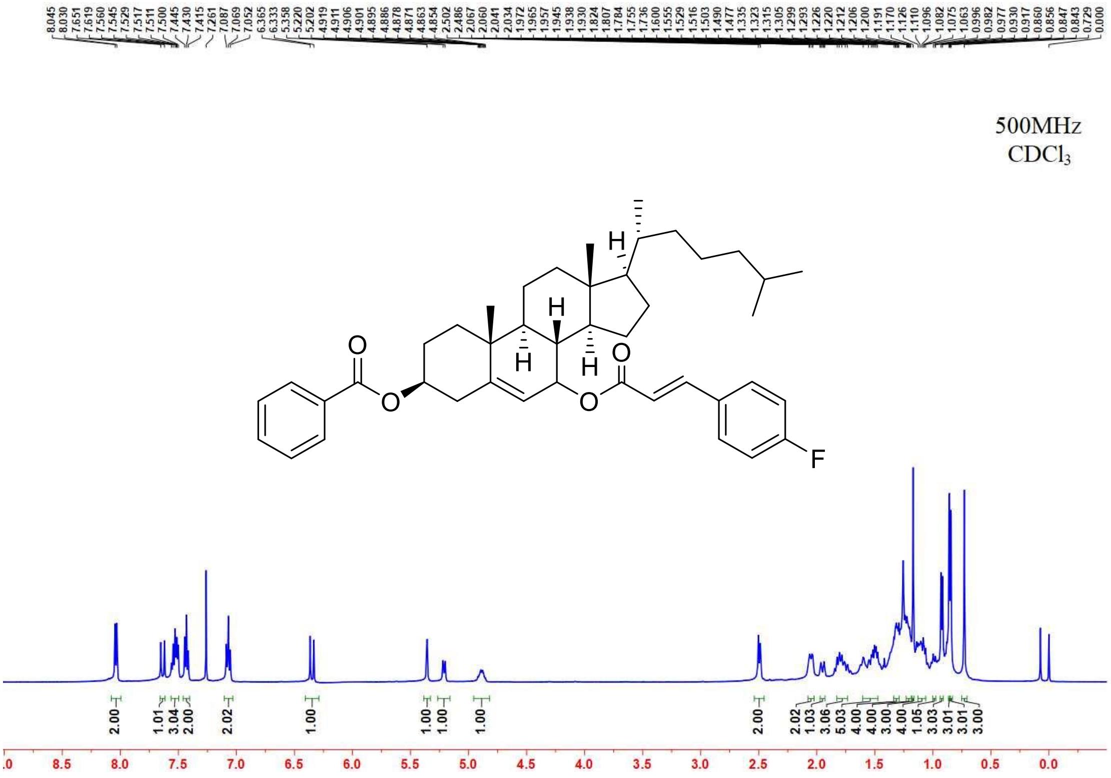

compound ***Ie***

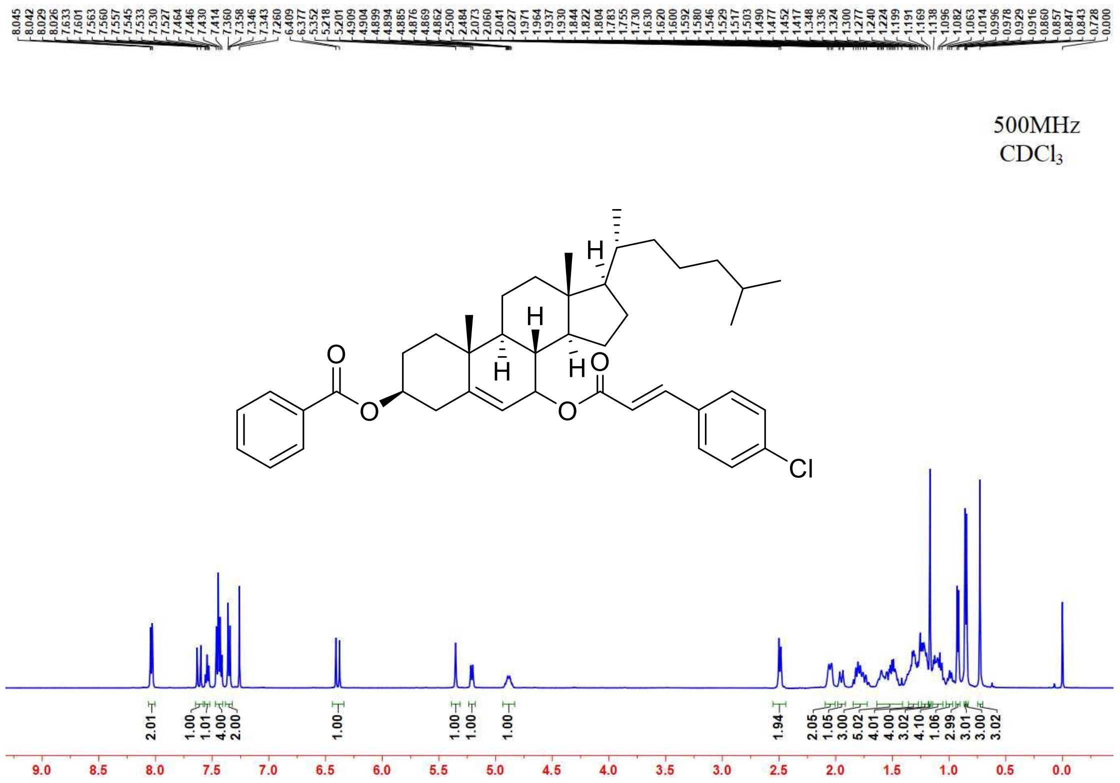

compound ***If***

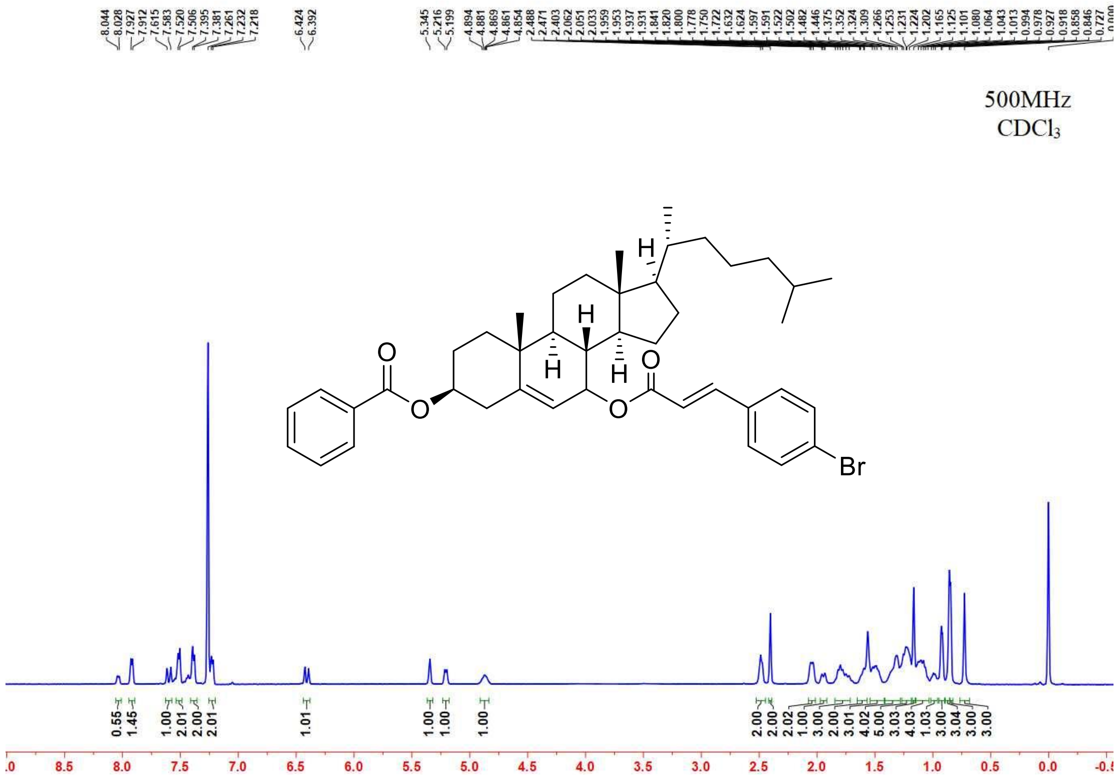

compound ***Ig***

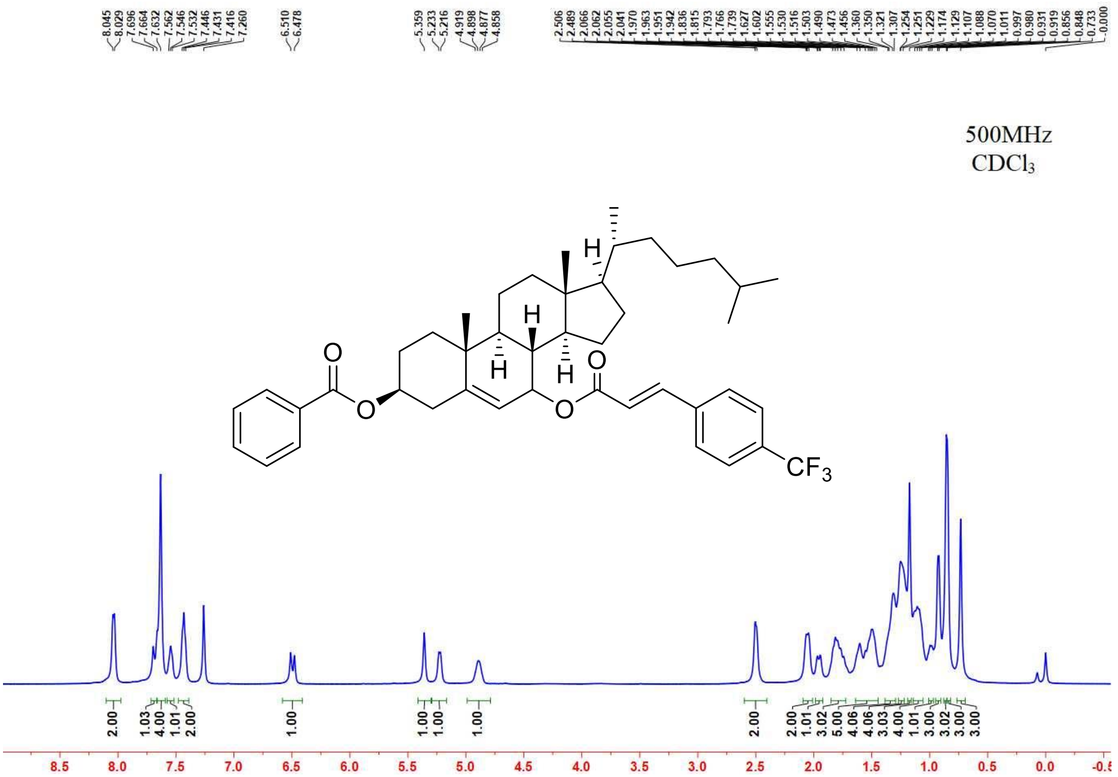

compound ***Ih***

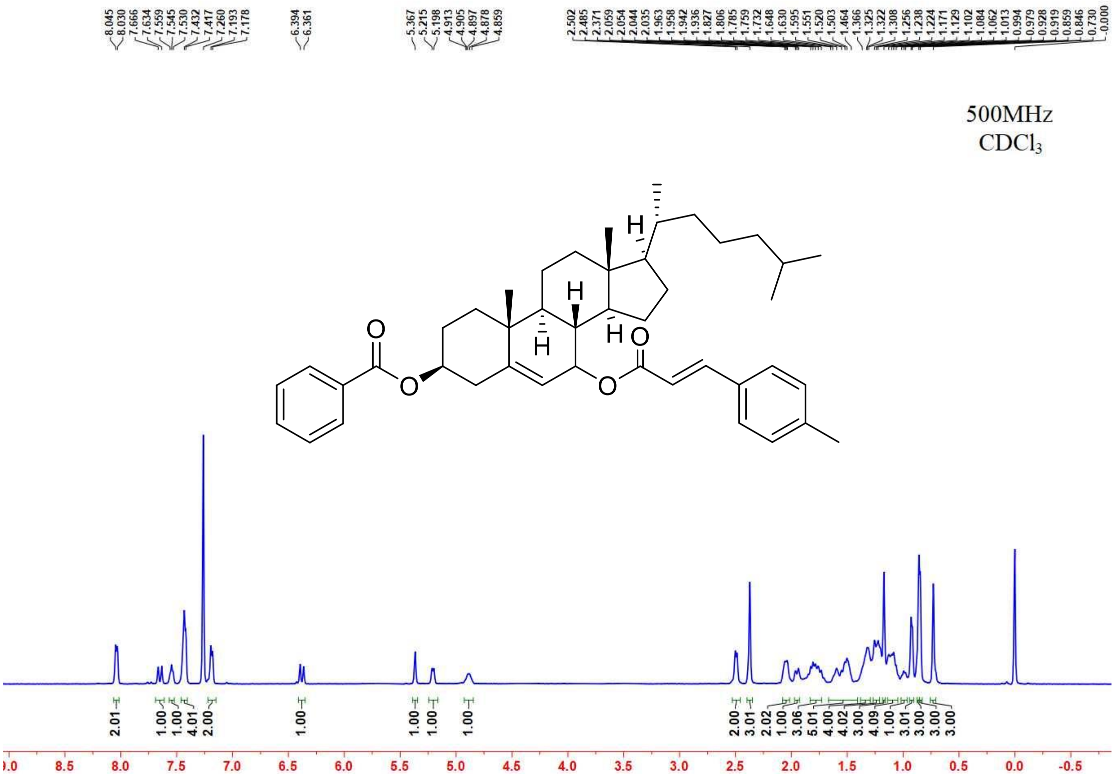

compound ***Ii***

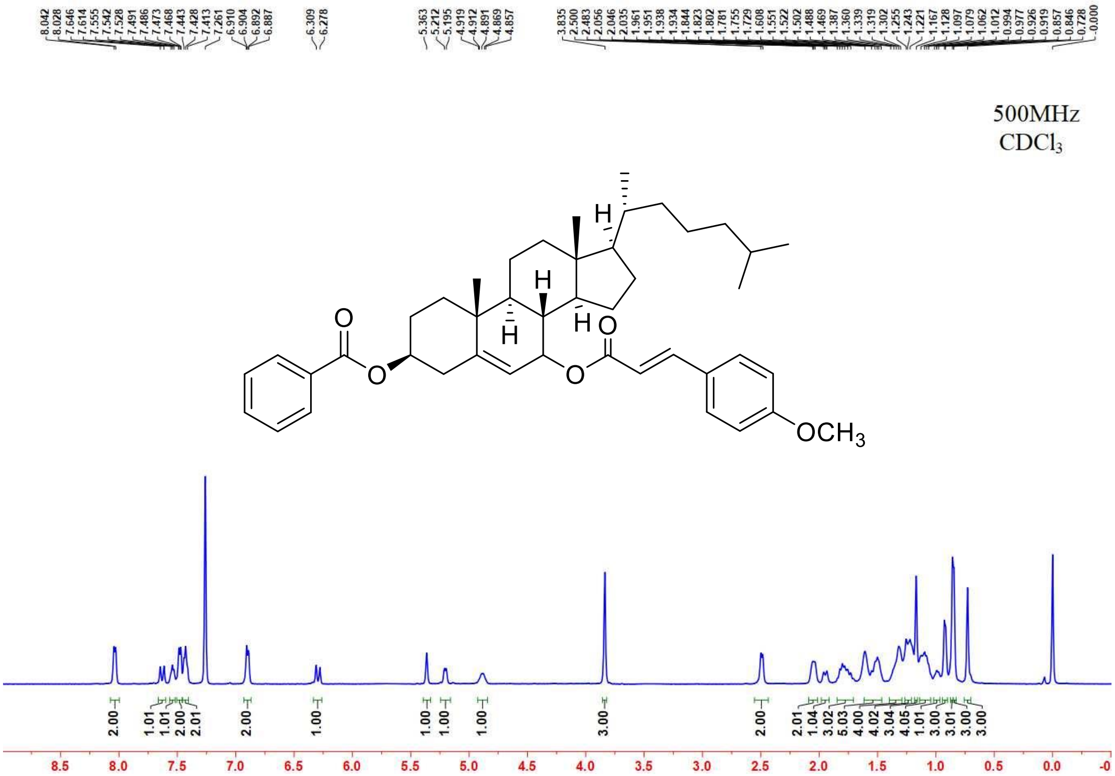

compound ***IIa***

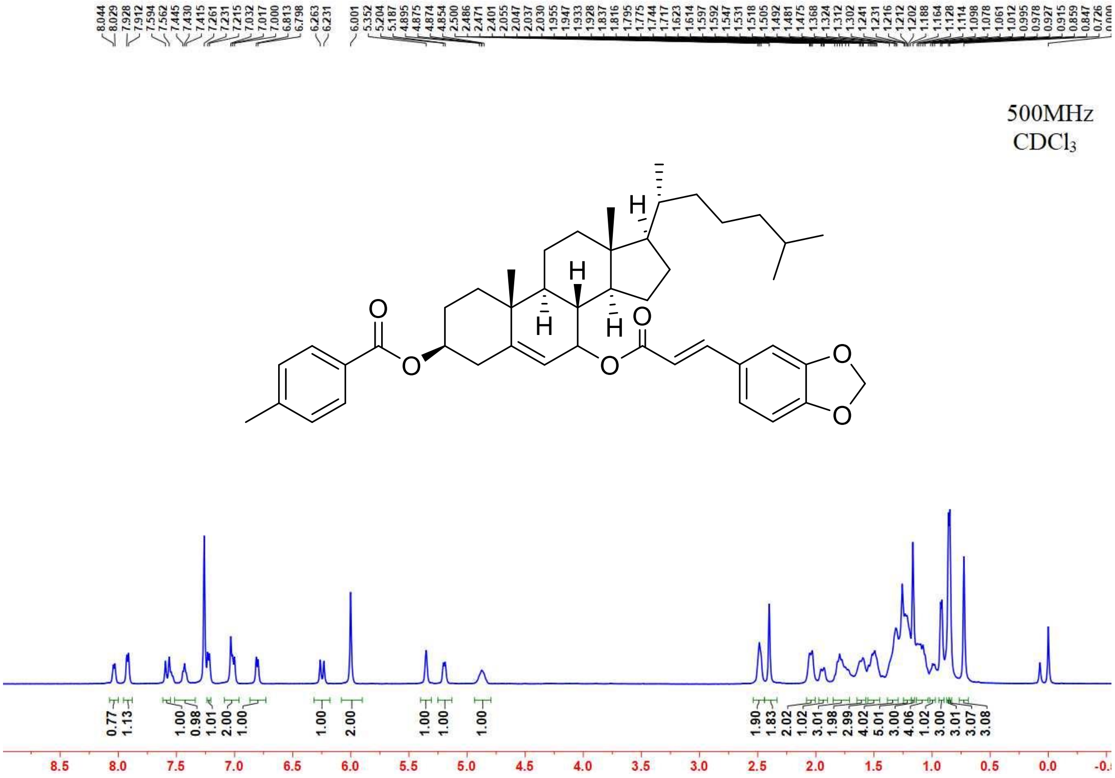

compound ***IIb***

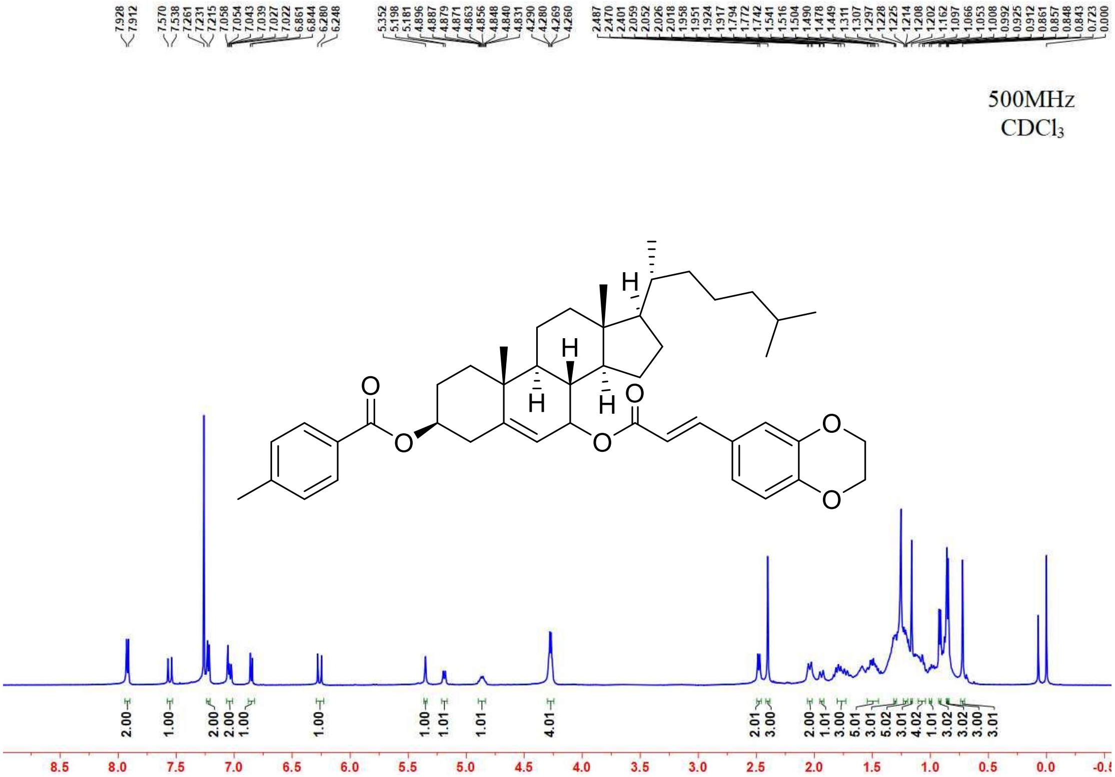

compound ***IIc***

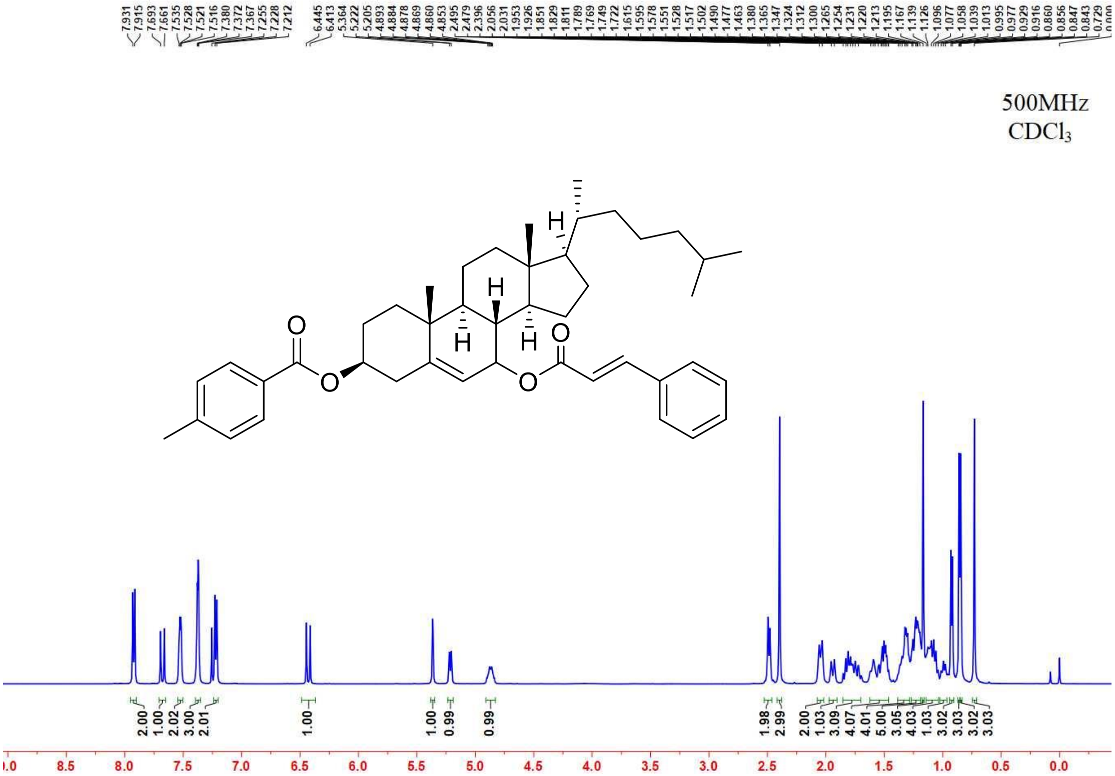

compound ***IId***

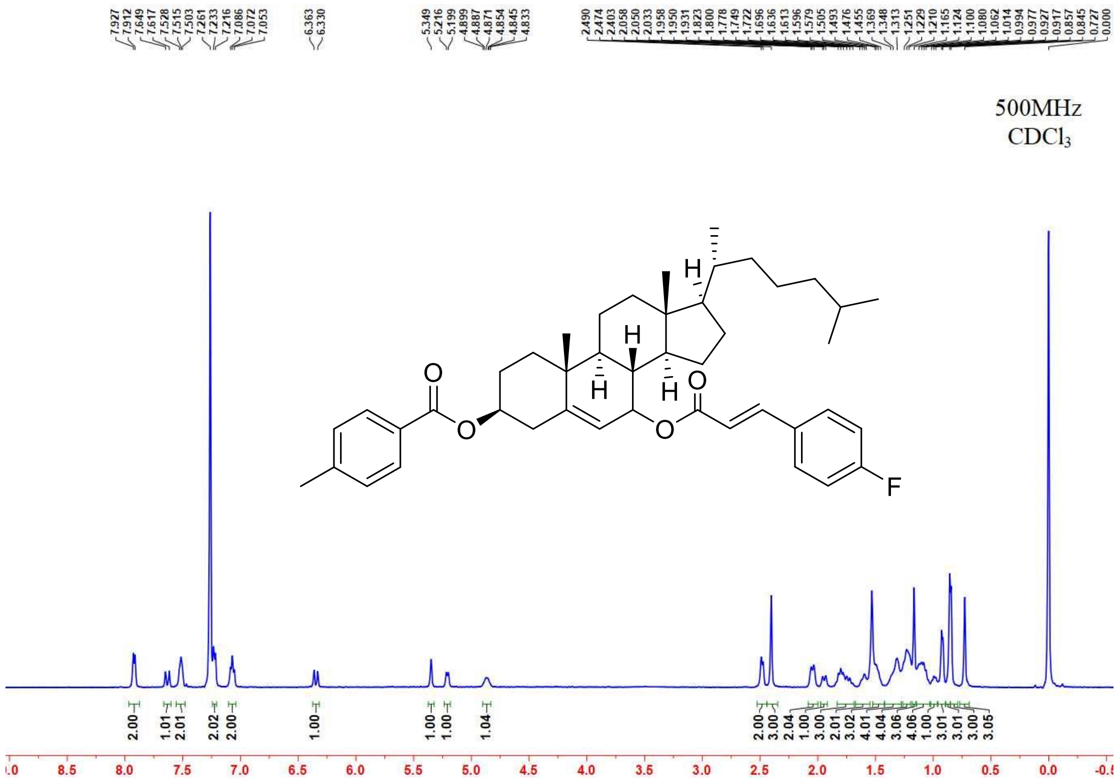

compound ***IIe***

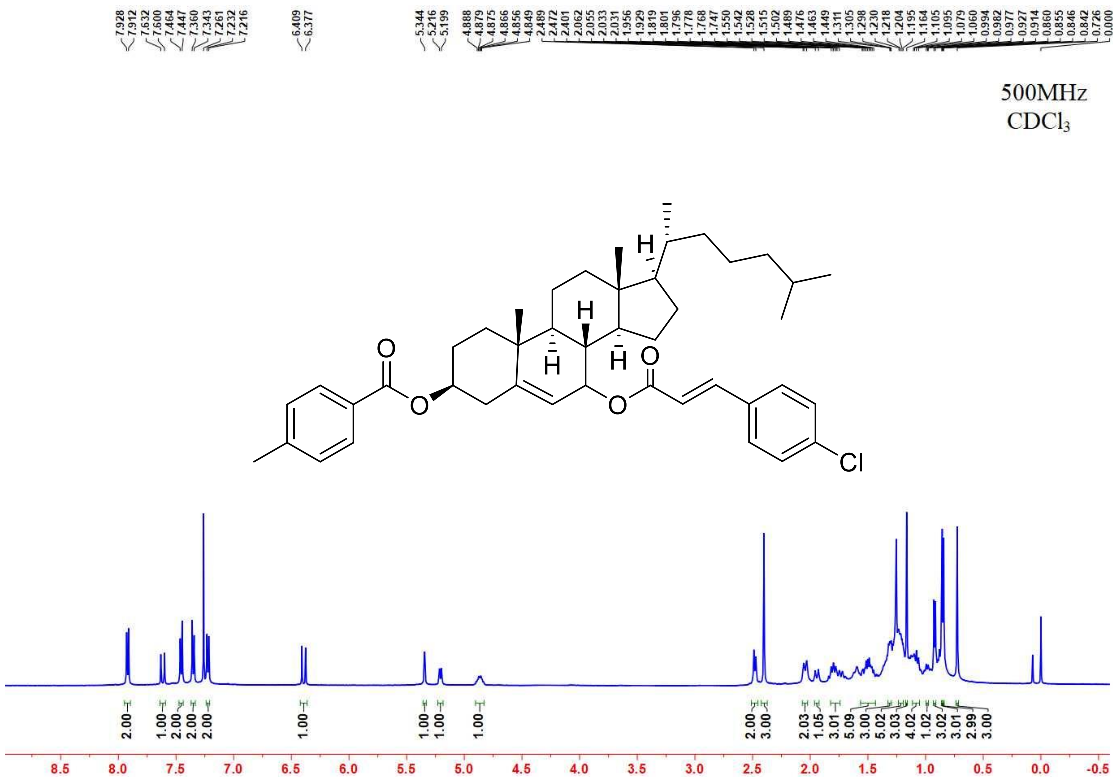

compound ***IIf***

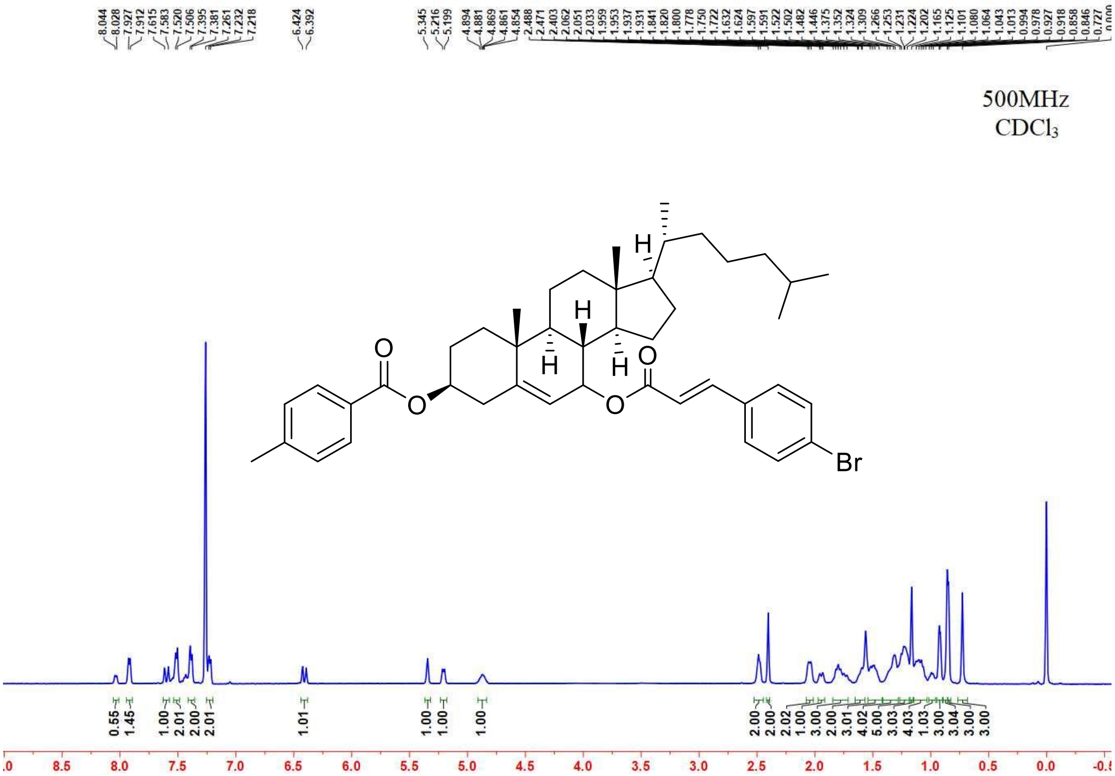

compound ***IIg***

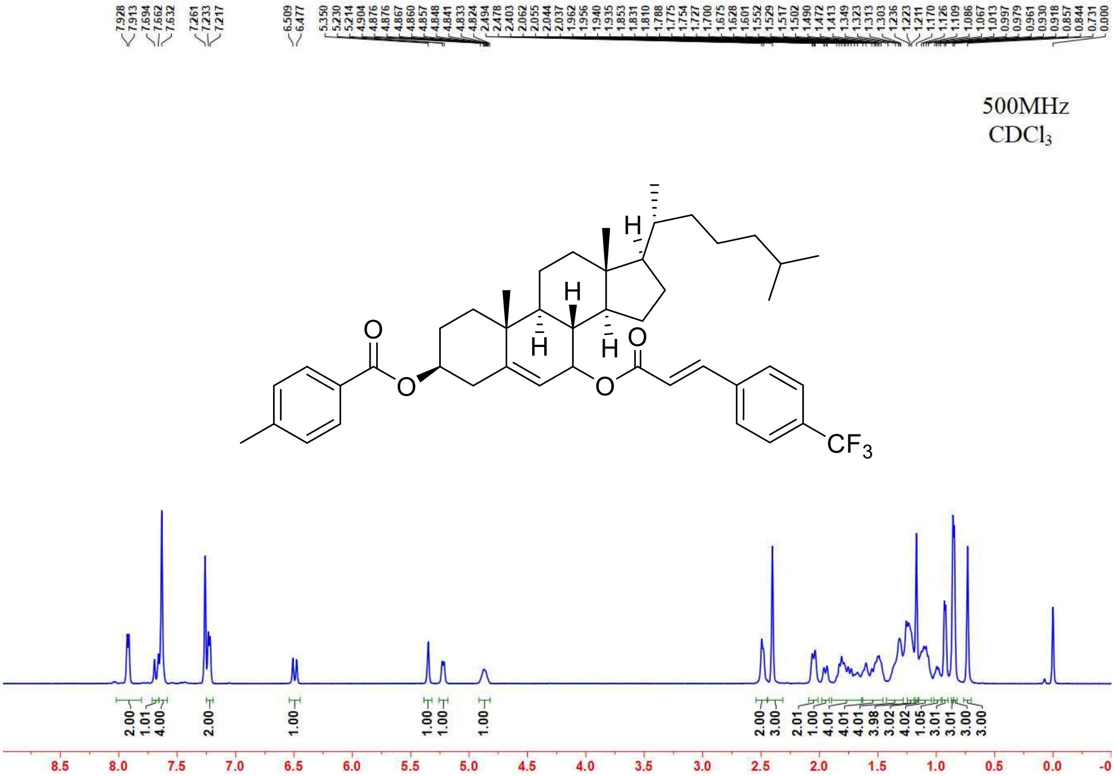

compound ***IIh***

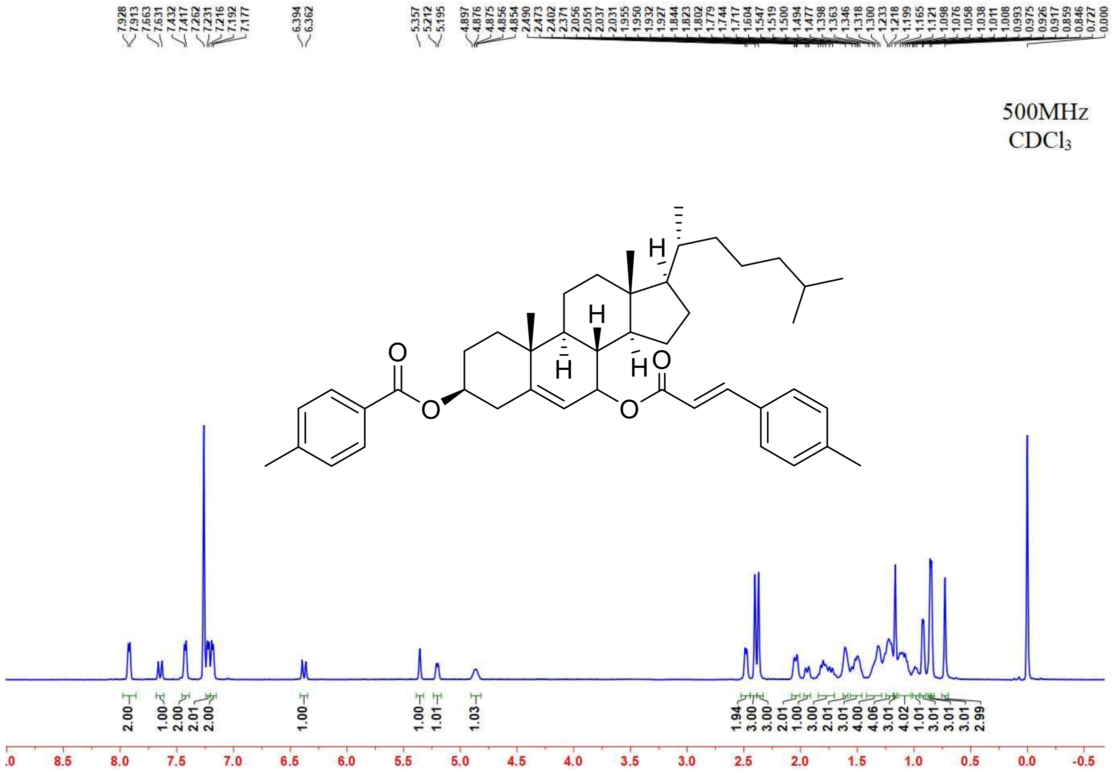

compound ***IIi***

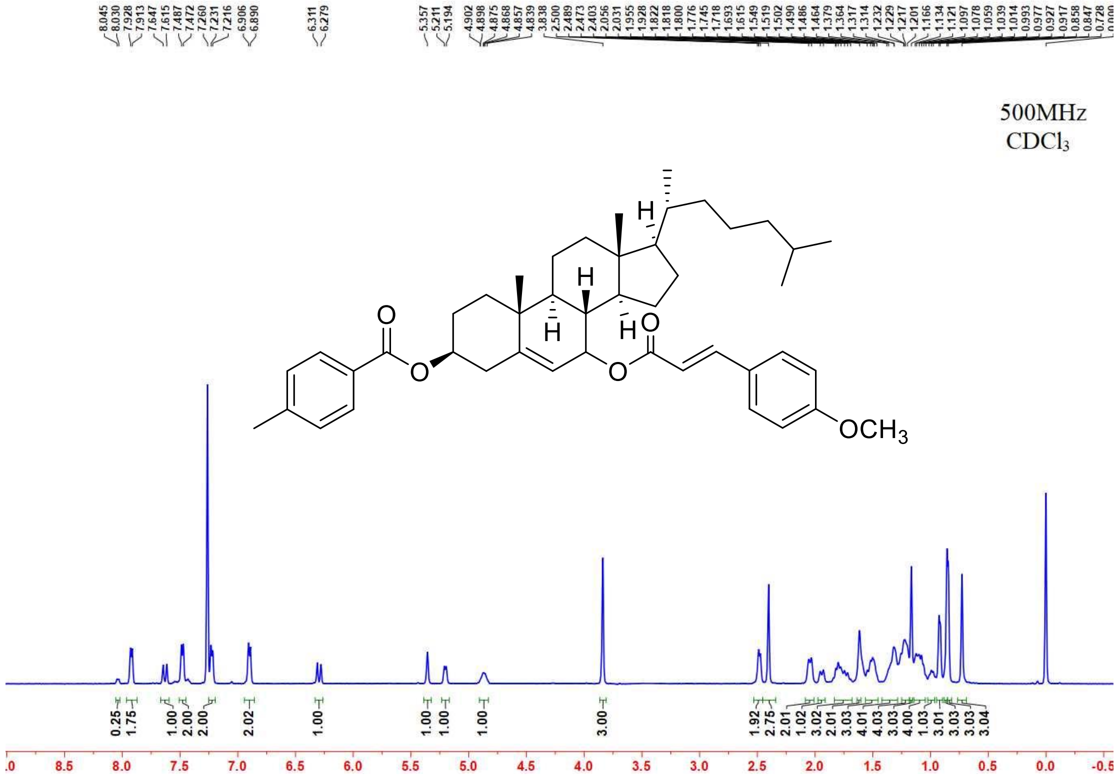

Supplement: Supplementary file 1 [file molecules-27-08437-s001.zip › molecules-2025334-supplementary.pdf]
